# Plasma One-Carbon Metabolism-Related Micronutrients and the Risk of Breast Cancer: Involvement of DNA Methylation

**DOI:** 10.3390/nu15163621

**Published:** 2023-08-17

**Authors:** Fubin Liu, Huijun Zhou, Yu Peng, Yating Qiao, Peng Wang, Changyu Si, Xixuan Wang, Jianxiao Gong, Kexin Chen, Fangfang Song

**Affiliations:** Department of Epidemiology and Biostatistics, Key Laboratory of Molecular Cancer Epidemiology, Key Laboratory of Prevention and Control of Major Diseases in the Population, Ministry of Education, National Clinical Research Center for Cancer, Tianjin Medical University Cancer Institute and Hospital, Tianjin Medical University, Tianjin 300060, China; liufubin826@tmu.edu.cn (F.L.); zhouhuijun0513@tmu.edu.cn (H.Z.); pengyu0629@tmu.edu.cn (Y.P.); qiaoyating@tmu.edu.cn (Y.Q.); wangpeng0510@tmu.edu.cn (P.W.); sichangyu@tmu.edu.cn (C.S.); wangxixuan@tmu.edu.cn (X.W.); gongjianxiao@tmu.edu.cn (J.G.)

**Keywords:** one-carbon metabolism, micronutrients, DNA methylation, breast cancer

## Abstract

Findings of epidemiologic studies focusing on the association between one-carbon metabolism-related micronutrients and breast cancer risk, along with the involvement of DNA methylation, have been inconsistent and incomprehensive. We conducted a case–control study in China including 107 paired participants and comprehensively detected 12 plasma one-carbon metabolism-related micronutrients. Genomic DNA methylation was measured using an 850 K chip and differential methylation probes (DMPs) were identified. Multivariate logistic regression was performed to estimate the associations between plasma micronutrients and the odds of breast cancer. The mediation of selected DMPs in micronutrient breast cancer associations was examined using mediation analyses. An inverse association of plasma folate, methionine cycling-related micronutrients (methionine, S-adenosylmethionine, and S-adenosylhomocysteine), and all micronutrients in the choline metabolism and enzymatic factor groups, and a positive association of methionine cycling-related cysteine with breast cancer risk were observed. Nine micronutrients (methionine, cysteine, SAM, folate, choline, betaine, P5P, vitamins B_2_, and B_12_) were related to global or probe-specific methylation levels (*p* < 0.05). The selected DMPs mediated the micronutrient breast cancer associations with an average mediation proportion of 36.43%. This study depicted comprehensive associations between circulating one-carbon metabolism-related micronutrients and breast cancer risk mediated by DNA methylation.

## 1. Introduction

Breast cancer is a serious threat to global women’s health. In 2020, approximately 2.26 million new cases and 680 thousand new deaths from breast cancer occurred worldwide [1]. Due to longer life expectancy, urbanization, and the adoption of Western foods and lifestyles, the incidence of breast cancer has increased significantly in developing countries [2]. As a public health problem, breast cancer will reduce the long-term quality of life of patients and bring heavy economic and social burdens [3]. Therefore, identifying modifiable risk factors of breast cancer to improve strategies for risk prevention is essential and urgent.

Dietary factors and nutritional status play an important role in the occurrence and development of breast cancer. Epidemiological studies have shown that the intake of dietary micronutrients involved in the one-carbon metabolism pathway influenced the onset and progression of breast cancer [4,5,6,7,8,9,10]. One-carbon metabolism is a universal metabolic process in cells in which organic groups containing one-carbon atoms are transferred to participate in nucleotide (e.g., purine and thymidylate) biosynthesis, providing methyl groups for all biological methylation reactions. This process is particularly important in the field of cancer metabolism [11]. Two major components of one-carbon metabolism comprise the folate cycle and methionine cycle involving choline metabolism and the participation of enzymatic factors (certain B vitamins) [12]. Some meta-analyses and observational studies have revealed an inverse association between some individual dietary one-carbon metabolism-related micronutrients (e.g., folate, choline, vitamins B2, and B6) and the risk of breast cancer [4,5,9]. Actually, the circulating level in the blood is a better indicator of the body’s overall metabolic load of nutrients than dietary intake. However, inconsistent findings of single circulating one-carbon metabolism-related micronutrients with breast cancer risk remain inconclusive [4,13,14,15,16,17,18,19,20,21,22]. To date, no study has comprehensively depicted an overview of circulating micronutrients in one-carbon metabolism (folate cycle, methionine cycle, choline metabolism, and enzymatic factors) as a functional set in relation to breast cancer risk.

DNA methylation is the most common and important form of epigenetic modification in breast cancer. The epigenome-wide association (EWAS) studies have shown a hypomethylation pattern of peripheral blood genomic DNA existing in breast cancer patients [23,24]. The micronutrients involved in one-carbon metabolism play a crucial part in maintaining genomic stability and methylation patterns [12]. Deficiency or excess of these micronutrients can affect the one-carbon metabolism process, change the availability of S-adenosylmethionine (SAM) in the methionine cycle, interfere with DNA and histone methylation patterns, and further lead to the occurrence and development of breast cancer [12]. Evidence suggested that some B vitamins appeared to be epigenetically active as they are involved in promoter methylation of breast cancer-related genes [25]. However, human data on whether DNA methylation is influenced by one-carbon metabolism-related micronutrients and mediates the association between these micronutrients and breast cancer are limited and inconsistent.

Herein, we aimed to conduct a hospital-based case–control study among Chinese women to examine the association of multiple plasma one-carbon metabolism-related micronutrients with the risk of breast cancer and DNA methylation, and further explore the mediation of DNA methylation in these biochemically-related micronutrients-breast cancer associations.

## 2. Materials and Methods

### 2.1. Study Population

The study population consisted of 107 paired breast cancer patients and controls, simply randomly extracted from a hospital-based case–control study we set up [26]. As described in our previous study [27], the cases were patients with newly diagnosed and histologically confirmed breast cancer from Tianjin Medical University Cancer Institute and Hospital since 1 January 2003. They had no history of other malignancies, no reception of any treatment before admission, and no history of blood transfusion in the last 6 months prior to blood sample collection. The age-matched (±1 year) healthy controls were the population without a history of malignancies who received health examination in the community during the same period. The process and purpose of the study were shown in Figure S1.

All participants enrolled in this study were of Chinese Han ethnicity. The Ethics Committee of Tianjin Medical University Cancer Institute and Hospital approved the study protocol (Ek2022212), and written informed consent was obtained from all patients and controls to participate in this study.

### 2.2. Data and Sample Collection

Information of each subject about demographics (age, education, household income, job, height, and weight), menstrual and reproductive history (age of menarche and first pregnancy, number of pregnancies and live births, breastfeeding months, menopausal status, and history of abortion, birth control pills use, estrogen replacement therapy, and sterilization surgery), environmental exposures (passive smoking and negative events), lifestyles (smoking and alcohol drinking), and family history of cancer in first-degree relatives was obtained from a structured questionnaire. Regarding negative events, participants were questioned, “Have you ever suffered from a negative life event (e.g., miserable marriage, abortion, bereavement, unemployment, illness in the family, change for adaptation, etc.) within the last year that caused a pronounced and lasting negative emotional experience” in the questionnaire, and asked to answer “yes” or “no” according to their own experiences and feelings. Body mass index (BMI) was calculated as weight (kg) divided by the square of height (m). Information on breast density was obtained from imaging examinations.

Five to 10 mL of fasting peripheral blood samples were collected from all subjects. After quiescence for 1–2 h, the blood samples were centrifuged at 3000 rpm for 15 min. Genomic DNA from leukocytes was isolated by the standard phenol–chloroform extraction. The separated plasma and extracted genomic DNA stock solution were stored in the refrigerator at −80 °C for subsequent use.

### 2.3. Measurements of Plasma One-Carbon Metabolism-Related Micronutrients

The one-carbon metabolism included two major pathways, i.e., folate cycle and methionine cycle, involving choline metabolism and some enzymatic factors (Figure S2). Therefore, we considered 12 one-carbon metabolism-related micronutrients and grouped them into four categories: (1) The methionine cycle group, including methionine, cysteine, homocysteine, SAM, and S-adenosylhomocysteine (SAH); (2) the folate cycle group, including folate and 5-methyltetrahydrofolate (5-MTHF); (3) the choline metabolism group, including choline and betaine; and (4) the enzymatic factor group, including pyridoxal 5-phosphate (P5P, the bioactive form of vitamin B_6_), vitamin B_2_, and vitamin B_12_.

Targeted metabolomic analysis of one-carbon metabolism-related micronutrients in plasma samples was performed using validated liquid chromatography–tandem mass spectrometry (LC-MS/MS) methods [28,29]. Sample injection and separation were performed by a SHIMAZDU 20AXR LC System interfaced with a QTRAP5500+^®^ (AB Sciex, Framingham, MA, USA) [29]. The samples were analyzed following injection of 10 μL of extract on a Phexnomonex Biphenyl 2.6 μm, 3.0 × 50 mm maintained at 35 °C and eluted in a gradient with buffer A (100% water with 0.1% formic acid + 0.01% heptafluorobutyric acid) and buffer B (100% methanol with 0.1% formic acid + 0.01% heptafluorobutyric acid). The flow rate was 0.5 mL/min, with a gradient over a total run time of 7 min: 0.0–2.0 min, 10% B; 2.0–3.0 min, 70% B; 3.0–5.0 min, 100% B; and 5.0–7.0 min, 10% B [28]. Mass spectrum conditions: the electric spray ion source and the negative ion mode were used with the ion source parameter atomization voltage of 5500 (+), the auxiliary heater of 70.0, the ion source temperature at 500.0 °C, the curtain gas of 30.0 and the atomization gas of 50.0. Scanning mode was multiple reaction monitoring. All data were collected and analyzed using Analyst software (version 1.7). Stable isotopes were used for each metabolite to account for matrix effects related to ion–suppression, and the quantitative values obtained were expressed as μmol/L or nmol/L. The accuracy of all metabolites was 85–115%.

### 2.4. DNA Methylation Assay

The pattern and extent of DNA methylation in the samples were determined using an Illumina Infinium Methylation EPIC BeadChip Kit (850K chip). DNA samples were processed by a series of steps according to the manufacturer’s protocols, i.e., DNA denaturation, whole genome amplification, fragmentation, precipitation, resuspension, hybridization, washing, elongation, staining, and scanning processes, to obtain methylation signals. The extent of methylation was determined by the β value, which was calculated as β = M/(M + U + 100), where M and U represented the signal strength of methylation and unmethylation, respectively. The β values ranged from 0–1, indicating completely unmethylated to totally methylated DNA [30]. The global DNA methylation was calculated as the average β values of all CpGs targeted by the 850K chip.

### 2.5. Data Processing for DNA Methylation Array

After quality control, we retained 98 cases and 102 controls for subsequent methylation analysis. The raw data of DNA methylation intensity were analyzed using the “ChAMP” package (version 2.21.1) [31]. We read 865,918 probes completely and then removed 9492 probes with a detection *p*-value above 0.01, 6957 probes with a bead count < 3 in more than 10 samples, 2882 probes with non-CpG sites, 94,837 probes with single nucleotide polymorphisms, 11 probes that aligned to multiple locations, and 16,694 probes located on the X and Y chromosomes. The remaining 735,045 probes were used for further analysis. Furthermore, the beta-mixture quantile (BMIQ) normalization method was used to adjust the intensity distribution of the Infinium II probes to fit that of the Infinium I probes [32], and the ComBat method was used for the correction of multiple batch effects [33]. We used Kolmogorov–Smirnov test methods to compare the distribution of array-wide mean methylation signal between case and control groups [34].

Array-wide differential methylation analysis was carried out using the “limma” package implemented by “champ.DMP” function. A CpG site with |Δβ| > 0.2 (case vs. control) and an adjusted *p*-value less than 0.05 was defined as a differentially methylated probe (DMP). The heatmap with unsupervised clustering was performed to evaluate the differentiating capacity of DMPs.

We subsequently estimated candidate differentially methylated regions (DMRs) using the “Bumphunter” algorithm [35], where clusters were defined by adjacent CpGs sites spaced up to 500 bp, and 1000 bootstrap samples were used to generate a null distribution of regions. We used the 95% quantile as the cutoff value for candidate regions with at least 7 CpGs probes. Considering its conservative nature relative to Benjamini–Hochberg (BH) and false discovery rate (FDR) methods, family-wise error rate (FWER) was used to control false positive rates with a threshold of <0.001.

To identify the potential biological pathways that DMPs and DMRs are involved in, the Gene Ontology (GO) and Kyoto Encyclopedia of Genes and Genomes (KEGG) analyses were performed with the “RclusterProfiler” package (Version 4.4.4.) [36]. The significance threshold was set at *p* < 0.05.

### 2.6. Statistical Analysis

Continuous variables were compared using the student’s *t*-test and categorical variables were compared using the Chi-square test. Twelve one-carbon metabolism-related micronutrients were converted to Z scores and then compared by rank-sum tests. A logistic regression model was applied to estimate the odds ratios (ORs) and 95% confidence intervals (CIs) to assess the association between one-carbon metabolism-related micronutrients and breast cancer risk. Combining the results of the distribution of basic characteristics with those well-known risk factors for breast cancer reported in the literature, the model was adjusted for per capita monthly household income, education, body mass index (BMI), negative events, benign breast disease, family history of cancer, age of menarche, menopausal status, number of pregnancies, number of live births, breastfeeding months, and abortion as covariates. We further examined the association between these micronutrients and breast cancer risk stratified by menopausal status.

We investigated correlations of one-carbon metabolism-related micronutrients with array-wide mean methylation levels and selected DMPs (intersection of DMPs and DMRs analyses), with the threshold of <0.05 for *p*-values corrected by FDR and Bonferroni methods. To ascertain if DNA methylation mediated the association between one-carbon metabolism-related micronutrients and breast cancer, we carried out a mediation analysis using the “mediator” package with adjustment for the same covariates previously identified to evaluate the direct effects, indirect effects, and proportion of mediation by the selected DMPs.

All analyses were performed using SAS version 9.4 (SAS Institute, Cary, NC, USA) and R software (The R Foundation, http://www.r-project.org, accessed on 1 June 2023, version 4.2.3). A level of <0.05 for two-sided *p*-values was considered statistically significant.

## 3. Results

### 3.1. Characteristics of Study Population

The main characteristics of participants by case and control groups are shown in Table 1. The age of the study population was 53.3 ± 6.0 years. Compared to healthy controls, breast cancer cases tended to be poorly educated, obese, with a low-income level, and have a history of benign breast disease, family history of cancer, and negative events. The differences in the distribution of reproductive factors such as pregnancies, live births, breastfeeding months, and abortion between cases and controls were statistically significant.

### 3.2. Association between One-Carbon Metabolism-Related Micronutrients and Breast Cancer Risk

**Figure 1:**
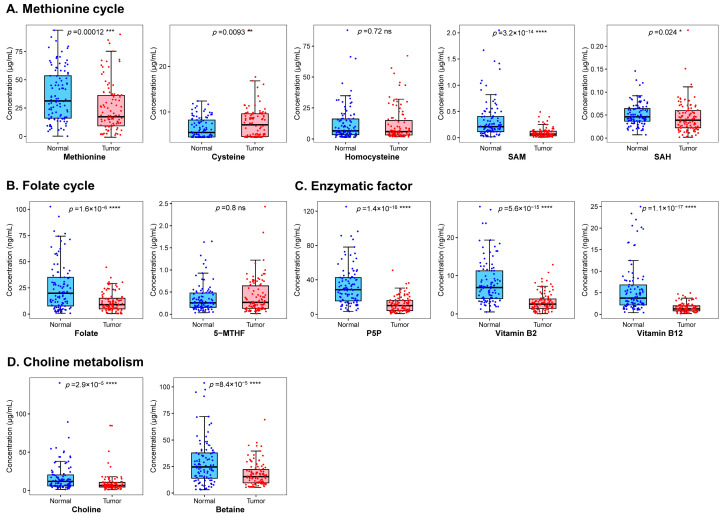
Comparison of plasma one-carbon metabolism-related micronutrients between case and control population. (**A**) Methionine cycle group, including methionine, cysteine, homocysteine, SAM, and SAH. (**B**) Folate cycle group, including folate and 5-MTHF. (**C**) Enzymatic factor group, including P5P and vitamins B_2_ and B_12_. (**D**) Choline metabolism group, including choline and betaine. The *p*-value represents the significance of rank-sum test. * <0.05; ** <0.01; *** <0.001; **** <0.0001. Abbreviations: 5-MTHF, 5-methyltetrahydrofolate; ns, not significant; P5P, pyridoxal 5-phosphate; SAH, S-adenosylhomocysteine; SAM, S-adenosylmethionine.

As shown in Figure 1, except for homocysteine and 5-MTHF, the concentrations of the other 10 one-carbon metabolism-related micronutrients were significantly different between cases and controls (*p* < 0.05), with the case group presenting lower levels of methionine, SAM and SAH in the methionine cycle group, folate, and all micronutrients in the enzymatic factor group and choline metabolism group, but a higher level of methionine cycling-related cysteine.

The univariate logistic regression analysis showed that 9 one-carbon metabolism-related micronutrients, i.e., methionine, SAM and SAH in the methionine cycle group, folate, and all micronutrients in the enzymatic factor group and choline metabolism group, were associated with a decreased risk of breast cancer, while a higher level of cysteine contributed to an increased breast cancer risk (Table 2). These significant effects remained in the model adjusted for covariates, and the ORs (95% CIs) was 0.59 (0.39–0.92) for methionine, 0.07 (0.02–0.23) for SAM, 0.60 (0.39–0.92) for SAH, 0.36 (0.18–0.70) for folate, 0.02 (0.004–0.17) for P5P, 0.09 (0.03–0.28) for vitamin B_2_, 0.01 (0.001–0.06) for vitamin B_12_, 0.43 (0.25–0.73) for choline, 0.36 (0.17–0.75) for betaine, and 1.79 (1.08–2.97) for cysteine, respectively.

Stratified analyses performed according to menopausal status are shown in Table 3. We found no multiplicative interactions between each of the micronutrients and menopausal status in breast cancer. In the fully-adjusted models, the levels of P5P and vitamin B_2_ had a similar trend of association with breast cancer risk, regardless of menopausal status. However, methionine was only associated with the risk of premenopausal breast cancer, but SAM, folate, vitamin B_12_, and choline were only linked to the risk of postmenopausal breast cancer.

### 3.3. Overall and Differential Analysis of DNA Methylation

**Figure 2:**
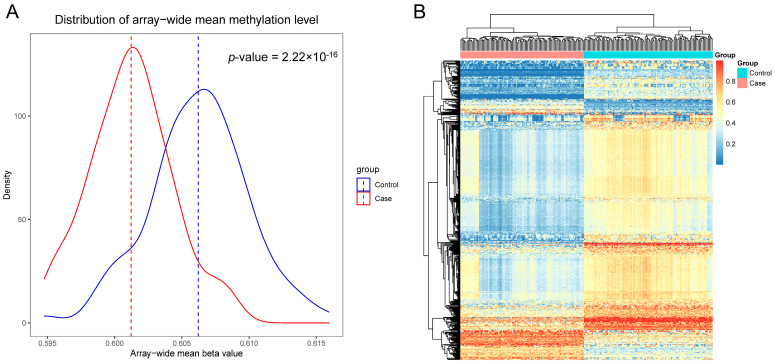
Overall and differential analysis of DNA methylation between breast cancer cases and control subjects. (**A**) Distribution of array-wide mean methylation level between breast cancer cases and control population. The *p*-value represents the significance of Kolmogorov–Smirnov test. (**B**) Heatmap of 524 differential methylation probes with unsupervised clustering.

We observed the global DNA methylation levels were significantly lower in the case group than that in the control group as tested by the Kolmogorov–Smirnov method (*p* = 2.2 × 10^−16^; Figure 2A). To further explore the DNA methylation differences between case and control subjects, we filtered the probes with a cut-off of |Δβ|-value > 0.2 and adjusted *p*-value of <0.05 by a two-group comparison and identified a total of 524 DMPs (Table S1), with a majority (85.3%, 447/524) exhibiting hypomethylation in the case group (Figure S3). Heatmap visualization with hierarchical clustering using these 524 DMPs showed two main clusters: one containing all cases, and the other containing all controls (Figure 2B).

With regard to the analysis of DMRs, we found 920 significant DMRs between case and control groups at a FWER < 0.001 (Table S2).

The GO pathway enrichment analyses revealed that the genes containing DMPs predominantly participated in one pathway, i.e., secretory granule membrane (Table S3). The 45 GO terms were significantly enriched in DMR analysis with the top 10 pathways representing biological processes such as embryonic development, neuron guidance, and cell adhesion (Table S4). No KEGG terms remained significant for DMPs and DMRs.

After an intersection of the sets of DMPs and DMRs based on genome location, 17 DMPs located in the DMRs were selected for further analysis (detailed in Table 4). Among these, most DMPs (88.2%, 15/17) were hypomethylated. Twelve DMPs were located in the promoter region (TSS200, TSS1500, and 1stExon) and five DMPs were located in CpG island.

### 3.4. Correlation between One-Carbon Metabolism-Related Micronutrients and DNA Methylation

**Figure 3:**
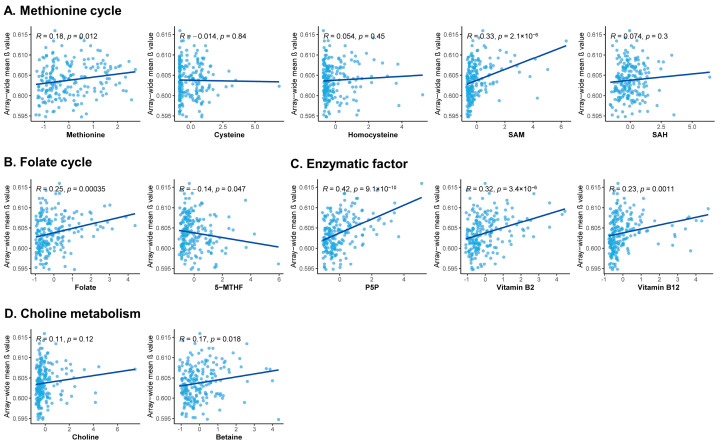
Correlation analysis between plasma one-carbon metabolism-related micronutrients and array-wide methylation levels. (**A**) Methionine cycle group, including methionine, cysteine, homocysteine, SAM, and SAH. (**B**) Folate cycle group, including folate and 5-MTHF. (**C**) Enzymatic factor group, including P5P, vitamins B_2_, and B_12_. (**D**) Choline metabolism group, including choline and betaine. The Spearman correlation coefficient and *p*-value are shown. Abbreviations: 5-MTHF, 5-methyltetrahydrofolate; P5P, pyridoxal 5-phosphate; SAH, S-adenosylhomocysteine; and SAM, S-adenosylmethionine.

We observed that methionine, SAM, folate, P5P, vitamin B2, vitamin B12, and betaine were positive, but 5-MTHF was negatively correlated with global methylation levels (Figure 3).

Of note is that among the aforementioned 17 selected DMPs, 3 DMPs (cg05397629, cg02589685, and cg10617037) can almost completely distinguish between cases and controls (Figure S4A–C) with area under curve values ≥ 0.999 (Figure S4D–F) and model accuracy ≥ 98.5% (Figure S4G–I). Therefore, they showed a misfit in multivariate logistic regression models for CpG and breast cancer in mediation analyses performed by the “mediator” R package and no results were received. Ultimately, those 3 DMPs were not included in the subsequent analysis. Thus, we explored the correlations between one-carbon metabolism-related micronutrients and 14 DMPs. As shown in Table S5, one-carbon metabolism-related micronutrients were related to some DMPs at a cut-off of 0.05 of unadjusted, FDR, and Bonferroni *p*-values. Under the Bonferroni standard, all the micronutrients, except for homocysteine, SAH, and 5-MTHF, were significantly related to DMPs, where SAM in the methionine cycle group, folate in the folate cycle group, betaine in the choline metabolism group, P5P, and vitamins B_2_ and B_12_ in the enzymatic factor group seemed more epigenetically active exhibiting the remarkable relationships with more than half of the DMPs.

### 3.5. Mediation of DNA Methylation in the Micronutrients-Breast Cancer Associations

**Figure 4:**
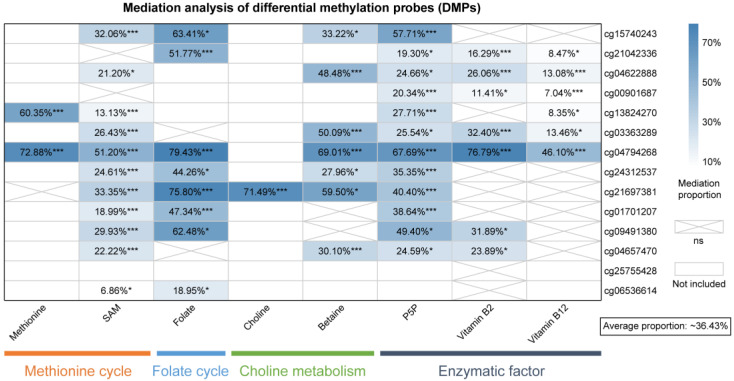
Mediation analysis of differential methylation probes (DMPs) in the association between plasma one-carbon metabolism-related micronutrients and risk of breast cancer. The mediation proportion (%) and significance of 14 DMPs for each one-carbon metabolism-related micronutrient are shown. The blank areas indicate that the micronutrients were not associated with the selected DMPs and thus not included in the mediation analysis. The areas marked with an “X” indicate an insignificant mediation proportion in the mediation analysis. * *p* < 0.05; *** *p* < 0.001. Abbreviations: ns, not significant; P5P, pyridoxal 5-phosphate; and SAM, S-adenosylmethionine.

Based on DMPs that were highly correlated with one-carbon metabolism-related micronutrients, the mediation analysis was conducted to examine the mediation of DMPs in the associations between one-carbon metabolism-related micronutrients and breast cancer risk (Figure 4 and Table S6). We observed an average mediation proportion of 36.43%. Specifically, the mediation proportion of DNA methylation was 6.86–72.88% for methionine cycling-related micronutrients including methionine and SAM, 18.95–79.43% for folate, 27.96–69.01% for choline metabolism-related micronutrients, and 19.30–76.79% for enzymatic factors, respectively. Only cg21697381 mediated the choline–breast cancer association. Moreover, the probe cg04794268 was the most widely used mediator in the associations between all micronutrients except choline and breast cancer with a mediation proportion of 46.10–79.43%.

## 4. Discussion

This case–control study among Chinese women suggested that higher plasma levels of B vitamins (folate, P5P, vitamins B_2_ and B_12_), and micronutrients involved in the methionine cycle (methionine, SAM, and SAH) and the choline metabolism (choline and betaine) were associated with a decreased risk of breast cancer, but cysteine level was positively associated with breast cancer risk. Most micronutrients were related to overall or probe-specific methylation levels. Moreover, DNA methylation to a larger extent mediated the micronutrient-breast cancer associations.

The methionine cycle is a major pathway of one-carbon metabolism, accompanied by the folate cycle and choline metabolism which are involved in homocysteine remethylation. The association between plasma homocysteine and the risk of breast cancer has been reported to be null in Western countries [15,16,17,18] and positive in China [14,19], the latter of which was consistent with our results. This signifies that race may be an important factor for inter-study heterogeneity. Population evidence for cysteine and breast cancer is mixed among American women [15,17,18,20]. We supported the positive association by utilizing a Chinese population. Actually, cysteine levels in our study were much lower (median levels were 5.61 μmol/L in controls) relative to those in other American studies (median levels were >200 μmol/L in controls). Discrepant findings among these studies may be attributable to the heterogeneity of the study populations with different exposure loads, whereas no studies based on human evidence were found on circulating levels of methionine, SAM, and SAH and their relation to breast cancer risk. Herein, we first documented inverse associations of these three micronutrients with breast cancer, where the association was more pronounced in premenopausal women for methionine and in postmenopausal women for SAM. A previous study revealed SAM inhibits the migration of triple-negative breast cancer cells in vitro [37], which may provide a theoretical basis for our findings. Epidemiological evidence from Western populations implied that regardless of menopausal status, the risk of breast cancer was contributed to a circulating level of 5-MTHF [13] but not folate [4,13,15,17]. Among Chinese populations, only an early study showed a negative but insignificant association between plasma folate and breast cancer risk [21]. Our results supported the negative association of plasma folate and no association of plasma 5-MTHF with breast cancer risk. The present study population has a higher concentration of folate and 5-MTHF than those in previous studies. Evidence suggested high intake of folate could saturate the activity of dihydrofolate reductase (DHFR), resulting in the presence of 5-MTHF and unmetabolized folate in the circulation [13]. Given this, our population might consume more dietary folate to overwhelm the activity of DHFR. However, the role of the *DHFR* gene in folic acid metabolism and breast cancer remains to be further investigated. Moreover, methylenetetrahydrofolate reductase (*MTHFR*) gene C677T (rs1081133) and A1298C (rs1801131) variants were reported to be factors predisposing breast cancer in Asians [38], but not in Western countries [15]. They may directly affect folate metabolite circulation levels to influence breast cancer onset and progression. For circulating choline and betaine, only a higher level of serum betaine contributed to a lower breast cancer risk in the Chinese population [22]. In the present study, we observed both plasma choline and betaine were protective factors for breast cancer, with the former being more pronounced in postmenopausal women. Taken together, more research is needed to clarify the role of one-carbon metabolism involving various functional groups in breast cancer.

Some other B vitamins, such as vitamins B_2_, B_6,_ and B_12_, are also key cofactors for one-carbon metabolism. The latest pooled analysis reported that a higher intake of dietary vitamin B2 might reduce the risk of breast cancer, but vitamins B_6_ and B_12_ do not [5]. A small number of studies suggested circulating B vitamins including PLP, vitamins B_2_ and B_12_ had no effects on breast cancer [5]. However, there is heterogeneity among studies and a lack of evidence on the Chinese population. We first reported inverse relationships between plasma levels of P5P and vitamins B_2_ and B_12_ with breast cancer risk in Chinese women. The effects of P5P and vitamin B_2_ were among both premenopausal and postmenopausal women and that of vitamin B_12_ was only among postmenopausal women. Nevertheless, more prospective studies and large-scale populations are warranted to support our results.

Hypermethylation of the promoter in cancer suppressor genes, accompanied by global hypomethylation of the genome, is a common feature of tumor cells [39]. Our results showed a genome-wide hypomethylation pattern in the breast cancer group, which was consistent with the previous EWAS studies of breast cancer using a 450 K chip [23,24]. We subsequently used 524 DMPs to form two independent clusters, suggesting the potential of DNA methylation as a classifier for cases and controls and as an optional biomarker in the future. Micronutrients in the one-carbon metabolism pathway have the ability to influence the epigenome and ultimately an individual’s risk of developing cancer [40]. The dietary methyl donors, such as folate, methionine, choline, and betaine, are able to modulate methylation patterns in animal models and humans [25,41]. Generally, we concluded that most one-carbon metabolism-related micronutrients (involving methyl donors and enzymatic factors) were related to array-wide and 14 probe-specific methylation levels. Additionally, we observed the associations between micronutrients from four groups and breast cancer more or less mediated by DNA methylation. To date, there is little evidence for a mediating role of DNA methylation in the association between these micronutrients and breast cancer. Nevertheless, our findings further clarified the possible involvement of DNA methylation in the process by which micronutrients involved in various parts of one-carbon metabolism influence the onset of breast cancer.

The hypomethylated probe cg04794268, located in the promoter of *KIFC2*, widely mediated the associations between almost all micronutrients and breast cancer in our study, although no report about the relationship between *KIFC2* and breast cancer has been found so far. Another interesting finding was the association of choline with a decreased risk of breast cancer only mediated by the hypomethylation of cg21697381, located in the open sea region of the *SLFN12* promoter. The over-expression of *SLFN12* can sensitize triple-negative breast cancer cells to chemotherapy drugs and radiotherapy [42]. However, whether the *SLFN12* gene is related to a reduced risk of breast cancer conferred by choline needs to be further investigated. Together, our findings make it interesting to systematically evaluate the role of DNA methylation modulation by one-carbon metabolism-related micronutrients in breast cancer in future studies.

To our knowledge, this was the first study to systematically consider one-carbon metabolism-related micronutrients as functional groups (methionine cycle, folate cycle, choline metabolism, and enzymatic factor) and comprehensively evaluate the potential role of plasma levels of these micronutrients in breast cancer and the involvement of DNA methylation in a Chinese population. Moreover, the whole genome methylation levels were first detected by an 850 K chip in a breast cancer study, providing wider coverage, higher data quality, and higher reproducibility. However, several limitations existing in the present study merited special consideration. Firstly, our study had the inherent limitations of a case–control study, such as recall bias and selection bias. Secondly, the main population in this study was citizens of Tianjin (a city in northern China), which may not be representative of the entire Chinese population. Thirdly, even though we had controlled the majority of confounders, the residual confounding from unmeasured or unknown factors might remain. Finally, due to the relatively small sample size of this study, the current analysis may be underpowered and the conclusions need to be verified by large-scale prospective data.

## 5. Conclusions

Our findings provided the first comprehensive depiction of an inverse association of plasma folate, micronutrients involved in the methionine cycle (methionine, SAM, and SAH) and choline metabolism (choline and betaine) and enzymatic factors in one-carbon metabolism (P5P, vitamins B_2_ and B_12_) and a positive association of methionine cycling-related cysteine with breast cancer risk in a Chinese population. Certain one-carbon metabolism-related micronutrients were related to array-wide and probe-specific methylation levels. Furthermore, differentially methylated CpGs partially or completely mediated the micronutrient-breast cancer associations. The above findings enrich the theoretical basis for the relationship between one-carbon metabolism-related micronutrients and individual methylation sites and provide evidence that one-carbon metabolism-related micronutrients may specifically affect breast cancer risk through different methylation sites. Meanwhile, one-carbon metabolism-related micronutrients may serve as a potential epigenetic drug to reverse abnormal methylation patterns in breast cancer, contributing to the development of targeted prevention and control measures for breast cancer. More prospective studies are needed in the future to clarify those associations, as well as to be motivated to uncover the specific mechanisms of action.

## Figures and Tables

**Table 1:** Baseline characteristics of 107 paired case and control population.

Variable	Group	Case (N = 107)	Control (N = 107)	*p*
Age, mean ± SD, years		53.3 ± 6.3	53.3 ± 5.8	0.991
Education, N (%)	Senior high school or above	55 (55.0)	66 (61.7)	0.043
	Junior high school	30 (30.0)	36 (33.6)	
	Primary school and below	15 (15.0)	5 (4.7)	
Per capita monthly household income, N (%), CNY	<500	4 (4.1)	2 (1.9)	<0.001
	500–999	15 (15.5)	6 (5.7)	
	1000–1999	47 (48.5)	32 (30.2)	
	2000–2999	20 (20.6)	33 (31.1)	
	≥3000	11 (11.3)	33 (31.1)	
Job, N (%)	No	53 (54.6)	55 (52.9)	0.803
	Yes	44 (45.4)	49 (47.1)	
BMI, N (%), kg/m^2^	<23.9	35 (32.7)	55 (51.9)	0.001
	24.0–27.9	46 (43.0)	43 (40.6)	
	≥28.0	26 (24.3)	8 (7.5)	
Benign breast disease, N (%)	No	66 (65.3)	83 (82.2)	0.007
	Yes	35 (34.7)	18 (17.8)	
Family history of cancer, N (%)	No	66 (62.3)	79 (75.2)	0.042
	Yes	40 (37.7)	26 (24.8)	
Family history of breast cancer, N (%)	No	97 (94.2)	104 (99.0)	0.087
	Yes	6 (5.8)	1 (1.0)	
Smoking, N (%)	No	101 (97.1)	97 (96.0)	0.673
	Yes	3 (2.9)	4 (4.0)	
Passive smoking, N (%)	No	33 (41.3)	31 (55.4)	0.106
	Yes	47 (58.8)	25 (44.6)	
Alcohol drinking, N (%)	No	102 (98.1)	100 (98.0)	0.984
	Yes	2 (1.9)	2 (2.0)	
Negative events, N (%)	No	74 (75.5)	89 (89.9)	0.008
	Yes	24 (24.5)	10 (10.1)	
Age of menarche, N (%), years	≤13	27 (26.0)	18 (17.1)	0.115
	14–15	41 (39.4)	39 (37.1)	
	≥16	36 (34.6)	48 (45.7)	
Number of pregnancies, N (%)	≥3	53 (51.0)	27 (25.7)	<0.001
	2	38 (36.5)	35 (33.3)	
	≤1	13 (12.5)	43 (41.0)	
Age of first pregnancy, N (%), years	<30	87 (87.9)	86 (85.1)	0.572
	≥30	12 (12.1)	15 (14.9)	
Number of live births, N (%)	≥2	30 (29.1)	13 (12.4)	0.003
	≤1	73 (70.9)	92 (87.6)	
Breastfeeding months, N (%), months	>12	63 (63.0)	43 (43.0)	0.005
	≤12	37 (37.0)	57 (57.0)	
Abortion, N (%)	No	18 (17.3)	42 (41.2)	<0.001
	Yes	86 (82.7)	60 (58.8)	
Take birth control pills, N (%)	No	84 (84.8)	84 (82.4)	0.633
	Yes	15 (15.2)	18 (17.6)	
Estrogen replacement therapy, N (%)	No	87 (89.7)	85 (94.4)	0.232
	Yes	10 (10.3)	5 (5.6)	
Sterilization surgery, N (%)	No	93 (91.2)	83 (91.2)	0.994
	Yes	9 (8.8)	8 (8.8)	
Menopausal status, N (%)	Premenopausal	34 (32.1)	33 (32.0)	0.995
	Postmenopausal	72 (67.9)	70 (68.0)	
Breast density, N (%)	Low	72 (75.8)	51 (63.0)	0.064
	High	23 (24.2)	30 (37.0)	

Abbreviations: BMI, body mass index; SD, standard deviation.

**Table 2:** Association of plasma one-carbon metabolism-related micronutrients with risk of breast cancer.

Group	Micronutrients	Median (P25, P75)		OR (95% CI)	
		Case (N = 107)	Control (N = 107)	Crude	Adjusted
Methionine cycle	Methionine, μmol/L	18.06 (9.27,37.02)	31.33 (15.50,55.15)	0.62 (0.46–0.83)	0.59 (0.39–0.92)
	Cysteine, μmol/L	6.95 (4.63,9.57)	5.61 (4.53,8.45)	1.44 (1.08–1.91)	1.79 (1.08–2.97)
	Homocysteine, μmol/L	4.91 (3.13,9.77)	6.47 (3.38,16.25)	0.75 (0.49–1.13)	0.84 (0.51–1.40)
	SAM, μmol/L	0.07 (0.04,0.13)	0.20 (0.11,0.37)	0.12 (0.06–0.26)	0.07 (0.02–0.23)
	SAH, μmol/L	0.04 (0.02,0.06)	0.05 (0.04,0.07)	0.70 (0.53–0.94)	0.60 (0.39–0.92)
Folate cycle	Folate, nmol/L	8.75 (4.79,15.35)	20.63 (8.37,36.57)	0.32 (0.21–0.50)	0.34 (0.18–0.70)
	5-MTHF, μmol/L	0.24 (0.13,0.57)	0.26 (0.16,0.47)	1.09 (0.83–1.42)	1.09 (0.72–1.65)
Enzymatic factor	P5P, nmol/L	9.94 (4.39,16.07)	28.55 (15.83,42.29)	0.10 (0.05–0.19)	0.02 (0.004–0.17)
	Vitamin B_2_, nmol/L	2.45 (1.39,3.85)	6.87 (3.67,11.34)	0.13 (0.07–0.24)	0.09 (0.03–0.28)
	Vitamin B_12_, nmol/L	1.23 (0.69,1.97)	3.76 (2.16,6.84)	0.02 (0.01–0.07)	0.01 (0.001–0.06)
Choline metabolism	Choline, μmol/L	6.58 (4.62,10.64)	11.84 (6.15,20.17)	0.33 (0.20–0.53)	0.43 (0.25–0.73)
	Betaine, μmol/L	15.26 (9.63,22.47)	23.83 (13.55,37.73)	0.42 (0.28–0.62)	0.36 (0.17–0.75)

Adjusted OR was adjusted for per capita monthly household income (CNY < 500, CNY 500–999, CNY 1000–1999, CNY 2000–2999, and CNY ≥ 3000); education (senior high school or above, junior high school, primary school and below); body mass index (<23.9 kg/m^2^, 24.0–27.9 kg/m^2^, ≥28.0 kg/m^2^); benign breast disease (no, yes); family history of cancer (no yes); negative events (no, yes); age of menarche (≤13 years, 14–15 years, and ≥16 years); menopausal status (premenopausal, postmenopausal); number of pregnancies (≥3, 2, ≤1); number of live births (≥2, ≤1); breastfeeding months (>12 months, ≤12 months); and abortion (no, yes). Abbreviations: 5-MTHF, 5-methyltetrahydrofolate; CI, confidence interval; OR, odds ratio; P5P, pyridoxal 5-phosphate; SAM, S-adenosylmethionine; and SAH, S-adenosylhomocysteine.

**Table 3:** Association of plasma one-carbon metabolism-related micronutrients with risk of breast cancer stratified by menopausal status.

Group	Micronutrients	Median (P25, P75)		OR (95% CI)	
		Case	Control	Crude	Adjusted
Premenopausal (N = 67)					
Methionine cycle	Methionine, μmol/L	18.62 (9.43,51.90)	41.85 (19.70,59.58)	0.62 (0.37–1.03)	0.07 (0.004–0.99)
	Cysteine, μmol/L	7.44 (4.62–9.80)	4.96 (4.48–6.91)	2.30 (1.20–4.40)	0.98 (0.39–2.46)
	Homocysteine, μmol/L	5.59 (3.09–18.97)	11.62 (4.66–18.97)	0.71 (0.42–1.18)	0.46 (0.08–2.55)
	SAM, μmol/L	0.09 (0.03–0.16)	0.17 (0.12–0.26)	0.16 (0.04–0.70)	0.19 (0.02–1.52)
	SAH, μmol/L	0.04 (0.02–0.05)	0.05 (0.04–0.07)	0.68 (0.41–1.12)	0.37 (0.08–1.72)
Folate cycle	Folate, nmol/L	7.86 (4.21–13.51)	11.80 (6.96–34.68)	0.36 (0.17–0.78)	0.21 (0.01–4.50)
	5-MTHF, μmol/L	0.20 (0.13–0.62)	0.24 (0.15–0.34)	1.42 (0.84–2.39)	0.32 (0.07–1.41)
Enzymatic factor	P5P, nmol/L	11.61 (4.80–18.32)	32.35 (16.66–50.75)	0.17 (0.06–0.43)	0.01 (0.00–0.82)
	Vitamin B_2_, nmol/L	2.81 (1.19–3.89)	6.18 (3.31–8.49)	0.18 (0.07–0.47)	0.01 (0.00–0.83)
	Vitamin B_12_, nmol/L	1.31 (0.73–2.01)	3.89 (2.45–8.00)	0.01 (0.00–0.10)	0.00 (0.00–6.33)
Choline metabolism	Choline, μmol/L	6.09 (4.03–8.76)	11.02 (5.98–23.63)	0.18 (0.05–0.63)	0.29 (0.05–1.71)
	Betaine, μmol/L	14.64 (9.74–23.77)	23.64 (16.75–37.16)	0.39 (0.19–0.81)	0.01 (0.00–1.44)
Postmenopausal (N = 143)					
Methionine cycle	Methionine, μmol/L	16.27 (9.14–34.30)	30.56 (15.37–51.28)	0.60 (0.42–0.86)	0.78 (0.38–1.59)
	Cysteine, μmol/L	6.95 (4.63–9.56)	6.06 (4.59–8.68)	1.21 (0.87–1.69)	1.97 (0.84–4.66)
	Homocysteine, μmol/L	4.91 (3.13–8.84)	4.95 (2.67–13.62)	0.86 (0.61–1.22)	1.03 (0.53–1.98)
	SAM, μmol/L	0.06 (0.04–0.11)	0.23 (0.11–0.47)	0.06 (0.02–0.19)	0.02 (0.002–0.21)
	SAH, μmol/L	0.04 (0.02–0.06)	0.05 (0.04–0.07)	0.73 (0.52–1.04)	0.72 (0.34–1.51)
Folate cycle	Folate, nmol/L	10.44 (4.98–16.50)	20.88 (10.98–40.04)	0.30 (0.17–0.53)	0.34 (0.13–0.92)
	5-MTHF, μmol/L	0.28 (0.13–0.46)	0.26 (0.16–0.48)	1.04 (0.74–1.45)	1.42 (0.69–2.91)
Enzymatic factor	P5P, nmol/L	8.98 (4.26–15.07)	25.49 (14.23–40.81)	0.08 (0.03–0.20)	0.02 (0.001–0.20)
	Vitamin B_2_, nmol/L	2.48 (1.42–3.94)	7.27 (4.15–12.65)	0.12 (0.05–0.26)	0.09 (0.02–0.53)
	Vitamin B_12_, nmol/L	1.18 (0.61–1.97)	3.29 (1.94–5.64)	0.09 (0.04–0.22)	0.01 (0.00–0.21)
Choline metabolism	Choline, μmol/L	7.39 (4.62–11.32)	11.73 (6.15–18.78)	0.54 (0.35–0.83)	0.36 (0.14–0.90)
	Betaine, μmol/L	15.60 (9.54–21.02)	23.85 (12.05–36.48)	0.53 (0.36–0.79)	0.58 (0.20–1.64)

All *p*-values for multiplicative interactions between micronutrients and menopausal status were >0.05. Adjusted OR was adjusted for per capita monthly household income (CNY < 500, CNY 500–999, CNY 1000–1999, CNY 2000–2999, and CNY ≥ 3000); education (senior high school or above, junior high school, primary school and below); BMI (<23.9 kg/m^2^, 24.0–27.9 kg/m^2^, ≥28.0 kg/m^2^); benign breast disease (no, yes); family history of cancer (no yes); negative events (no, yes); age of menarche (<13 years, 14–15 years, and ≥16 years); number of pregnancies (≥3, 2, ≤1); number of live births (≥2, ≤1); breastfeeding months (>12 months, ≤12 months); and abortion (no, yes). Abbreviations: 5-MTHF, 5-methyltetrahydrofolate; CI, confidence interval; OR, odds ratio; P5P, pyridoxal 5-phosphate; SAM, S-adenosylmethionine; and SAH, S-adenosylhomocysteine.

**Table 4:** Information on 17 selected differential methylation probes (DMPs).

CpG	Δβ	Chr	CpG Position	CpG Related Traits	Gene	Gene Position	Gene Molecular Function	Gene Pathways
cg05397629	−0.597	11	opensea	/	*RHOG*	Body	Nucleotide binding; GTPase activity	PI5P, PP2A, and IER3 regulate PI3K/AKT Signaling; signaling by Rho GTPases
cg02589685	−0.451	11	shore	/	/	IGR	/	/
cg03363289	−0.257	9	island	Breast cancer prognosis; colorectal cancer	*LHX6*	Body	DNA binding	/
cg04622888	−0.239	9	island	Gingivo-buccal oral squamous cell carcinoma; breast cancer prognosis; colorectal cancer	*LHX6*	TSS200	DNA binding	/
cg09491380	−0.234	4	opensea	/	*MAEA*	Body	Actin binding; ubiquitin protein transferase activity	Ciliary landscape
cg10617037	−0.229	8	shore	/	*CYHR1*	1stExon	Zinc ion binding	/
cg15740243	−0.229	5	shore	/	*RNF145*	TSS1500	Zinc ion binding; transferase activity	/
cg00901687	−0.218	17	shore	Aging; Alzheimer’s disease (AD)	*MYCBPAP*	TSS1500	Protein binding; phospholipid binding; clathrin binding	/
cg24312537	−0.215	8	shore	Crohn’s disease (CD); gestational diabetes mellitus; mortality	*HTRA4*	TSS1500	Serine-type endopeptidase activity; protein binding	/
cg06536614	−0.215	5	island	Breast cancer risk	*MIR886*	TSS200	/	/
cg04657470	−0.213	2	island	Tetralogy of Fallot	*HSPE1*	1stExon	RNA binding; protein binding; ATP binding	Signaling by Rho GTPases; RAC2 GTPase cycle
cg04794268	−0.208	8	shore	Early metastasis of uveal melanoma	*KIFC2*	TSS1500	Nucleotide binding; cytoskeletal motor activity	Golgi-to-ER retrograde transport; vesicle-mediated transport
cg21042336	−0.204	18	shore	/	*OSBPL1A*	TSS1500	Protein binding; phospholipid binding; lipid binding	Synthesis of bile acids and bile salts; metabolism
cg21697381	−0.203	17	opensea	B acute lymphoblastic leukemia	*SLFN12*	TSS1500	RNA nuclease activity; protein binding	17q12 copy number variation syndrome
cg01701207	−0.203	16	opensea	Allergic asthma	*SF3B3*	Body	Nucleic acid binding; protein binding	Processing of Capped Intron-Containing Pre-mRNA
cg25755428	0.229	19	island	/	*MRI1*	TSS1500	Protein binding; isomerase activity	Methionine de novo and salvage pathway; sulfur amino acid metabolism
cg13824270	0.270	6	shore	Breast cancer	*PRPF4B*	TSS1500	Nucleotide binding; RNA binding; protein kinase activity	Processing of Capped Intron-Containing Pre-mRNA

The information on CpG-related traits was obtained from EWAS Atlas (ngdc.cncb.ac.cn/ewas/atlas, accessed on 28 July 2023). The information on gene molecular function and gene pathways was obtained from GeneCards (www.genecards.org, accessed on 28 July 2023). Abbreviations: Chr, chromosome; IGR, intergenic region; TSS, transcription start site.

## Data Availability

The data used for this analysis can be made available upon reasonable request to the corresponding authors.

## Supplementary Materials

The following supporting information can be downloaded at: https://www.mdpi.com/article/10.3390/nu15163621/s1, Figure S1: flowchart of the study; Figure S2: overview of one-carbon metabolism and its related micronutrients; Figure S3: volcano plot of analysis of differential methylation probes (DMPs); Figure S4: details about distinguishing capacity of three differential methylation probes (DMPs); Table S1: information of 524 differentially methylation probes (DMPs); Table S2: information of 920 differentially methylation regions (DMRs); Table S3: gene ontology terms that were significantly enriched in an analysis of differentially methylation probes (DMPs); Table S4. gene ontology terms that were significantly enriched in an analysis of differentially methylation regions (DMRs); Table S5: correlation analysis between plasma one-carbon metabolism-related micronutrients and 14 differential methylation probes (DMPs); Table S6: mediation analysis of differential methylation probes (DMPs) in the association between plasma one-carbon metabolism-related micronutrients and risk of breast cancer.

## Abbreviations

5-MTHF, 5-methyltetrahydrofolate; BH, Benjamini–Hochberg; BMI, body mass index; BMIQ, beta-mixture quantile; CI, confidence interval; DHFR, dihydrofolate reductase; DMP, differentially methylated probe; DMR, differentially methylated region; EWAS, epigenome-wide association; FDR, false discovery rate; FWER, family-wise error rate; GO, Gene Ontology; KEGG, Kyoto Encyclopedia of Genes and Genomes; LC-MS/MS, liquid chromatography–tandem mass spectrometry; MTHFR, methylenetetrahydrofolate reductase; OR, odds ratio; P5P, pyridoxal 5-phosphate; SAH, S-adenosylhomocysteine; SAM, S-adenosylmethionine.
