# Supplementary material for: Plasma One-Carbon Metabolism-Related Micronutrients and the Risk of Breast Cancer: Involvement of DNA Methylation
Source: Nutrients. 2023 Aug 17;15(16):3621. doi: 10.3390/nu15163621 (PMC10458034; doi:10.3390/nu15163621)
Supplement: Supplementary file 1 [file nutrients-15-03621-s001.zip › Figures S1-S4 and Tables S3-S6.pdf]

*Supplementary Materials*

# Plasma One-Carbon Metabolism-Related Micronutrients and the Risk of Breast Cancer: Involvement of DNA Methylation

**Fubin Liu †, Huijun Zhou †, Yu Peng, Yating Qiao, Peng Wang, Changyu Si, Xixuan Wang, Jianxiao Gong, Kexin Chen * and Fangfang Song ***

Department of Epidemiology and Biostatistics, Key Laboratory of Molecular Cancer Epidemiology, Key Laboratory of Prevention and Control of Major Diseases in the Population, Ministry of Education, National Clinical Research Center for Cancer, Tianjin Medical University Cancer Institute and Hospital, Tianjin Medical University, Tianjin 300060, China; liufubin826@tmu.edu.cn (F.L.); zhouhuijun0513@tmu.edu.cn (H.Z.); pengyu0629@tmu.edu.cn (Y.P.); qiaoyating@tmu.edu.cn (Y.Q.); wangpeng0510@tmu.edu.cn (P.W.); sichangyu@tmu.edu.cn (C.S.); wangxixuan@tmu.edu.cn (X.W.); gongjianxiao@tmu.edu.cn (J.G.)

* Correspondence: chenkexin@tmu.edu.cn (K.C.); songfangfang@tmu.edu.cn (F.S.); Tel./Fax: +86-(0)2223372231 (F.S. & K.C.)

† These authors contributed equally to this work.

# Supplementary Materials

Figure S1. Flow chart of the study

Figure S2. Overview of one-carbon metabolism and its related micronutrients

Figure S3. Volcano plot of analysis of differential methylation probes (DMPs)

Figure S4. Details about distinguishing capacity of three differential methylation probes (DMPs)

Table S1. Information of 524 differentially methylation probes (DMPs)

Table S2. Information of 920 differentially methylation regions (DMRs)

Table S3. Gene ontology terms that were significantly enriched in an analysis of differentially methylation probes (DMPs)

Table S4. Gene ontology terms that were significantly enriched in an analysis of differentially methylation regions (DMRs)

Table S5. Correlation analysis between plasma one-carbon metabolism-related micronutrients and 14 differential methylation probes (DMPs)

Table S6. Mediation analysis of differential methylation probes (DMPs) in the association between plasma one-carbon metabolism-related micronutrients and risk of breast cancer

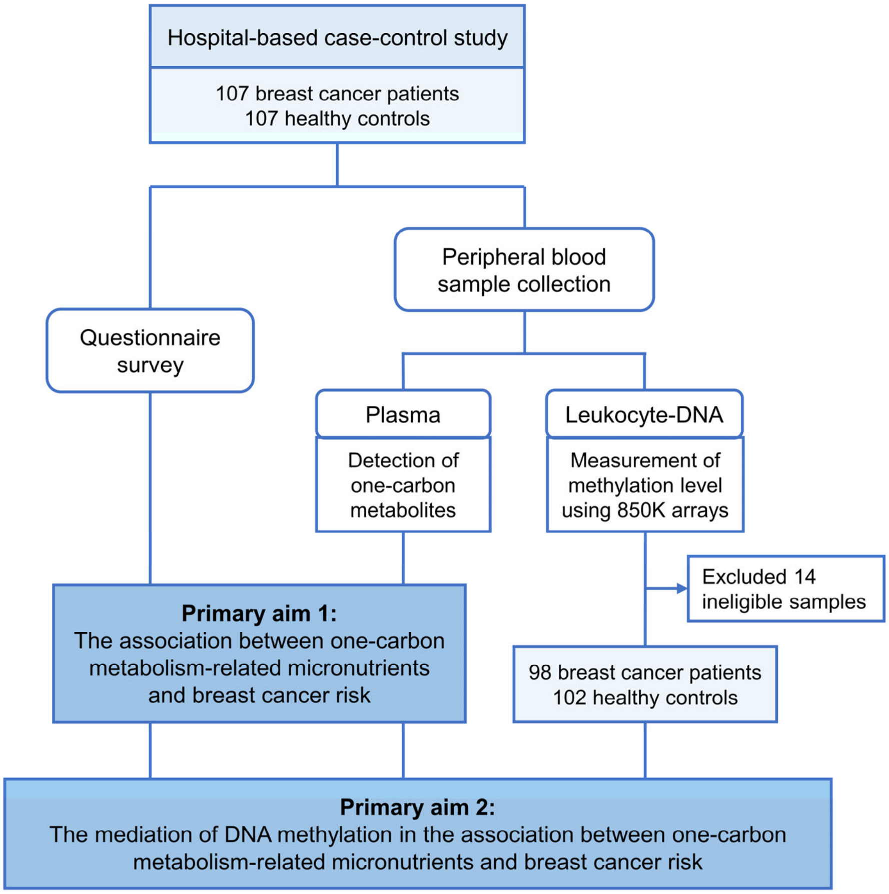


**Figure S1.** Flow chart of the study.

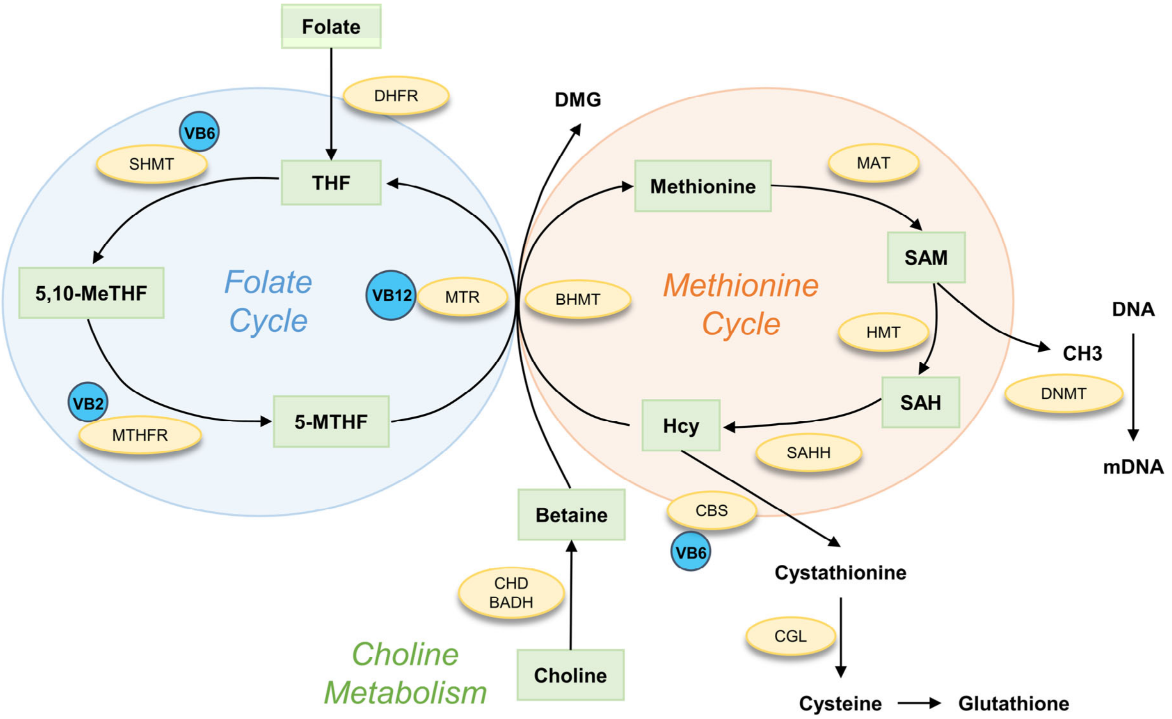


**Figure S2.** Overview of one-carbon metabolism and its related micronutrients. Two major components of one-carbon metabolism comprise the folate cycle and methionine cycle. The choline metabolism is mainly involved in the re-methylation of Hcy, parallel to the widespread folate-dependent homocysteine re-methylation pathway. The Vitamins B2, B6 and B12 participate in one-carbon metabolism as enzymatic factors. The SAM acts as a methyl-donor to provide methyl for DNA methylation process under the action of DNMT. Abbreviations: 5,10-MeTHF, 5,10-methylenetetrahydrofolate; 5-MTHF, 5-methyltetrahydrofolate; BADH, betaine aldehyde dehydrogenase; BHMT, betaine homocysteine methyltransferase; CBS, cystathionine β-synthase; CGL, Cystathionine γ-lyase; CHD, choline dehydrogenase; DHFR, dihydrofolate reductase; DMG, dimethylglycine; DNMT, DNA methyltransferase; Hcy, homocysteine; HMT, histone methyl transferase; MAT, methionine adenosyltransferase; MTHFR, methyltetrahydrofolate reductase; MTR, methionine synthase; SAH, S-adenosylhomocysteine; SAHH, S-adenosylhomocysteine hydrolase; SAM, S-adenosylmethionine; SHMT, serine hydroxymethyltransferase; THF, tetrahydrofolate; VB12, vitamin B12; VB2, vitamin B2; VB6, vitamin B6.

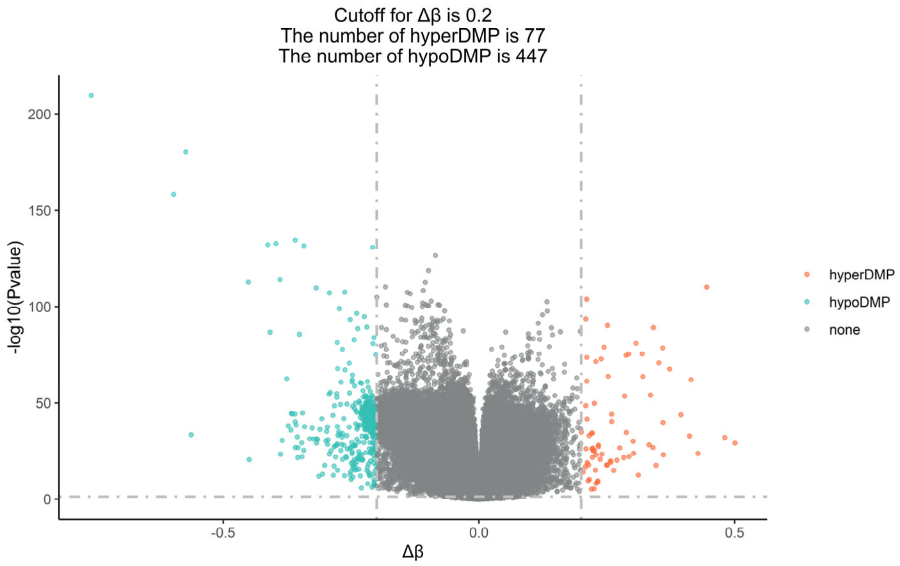


**Figure S3.** Volcano plot of analysis of differential methylation probes (DMPs). The 524 DMPs contained 77 hypermethylated and 447 hypomethylated DMPs using the cut-off value of adjusted *P*-value =0.05 and |Δβ| =0.2.

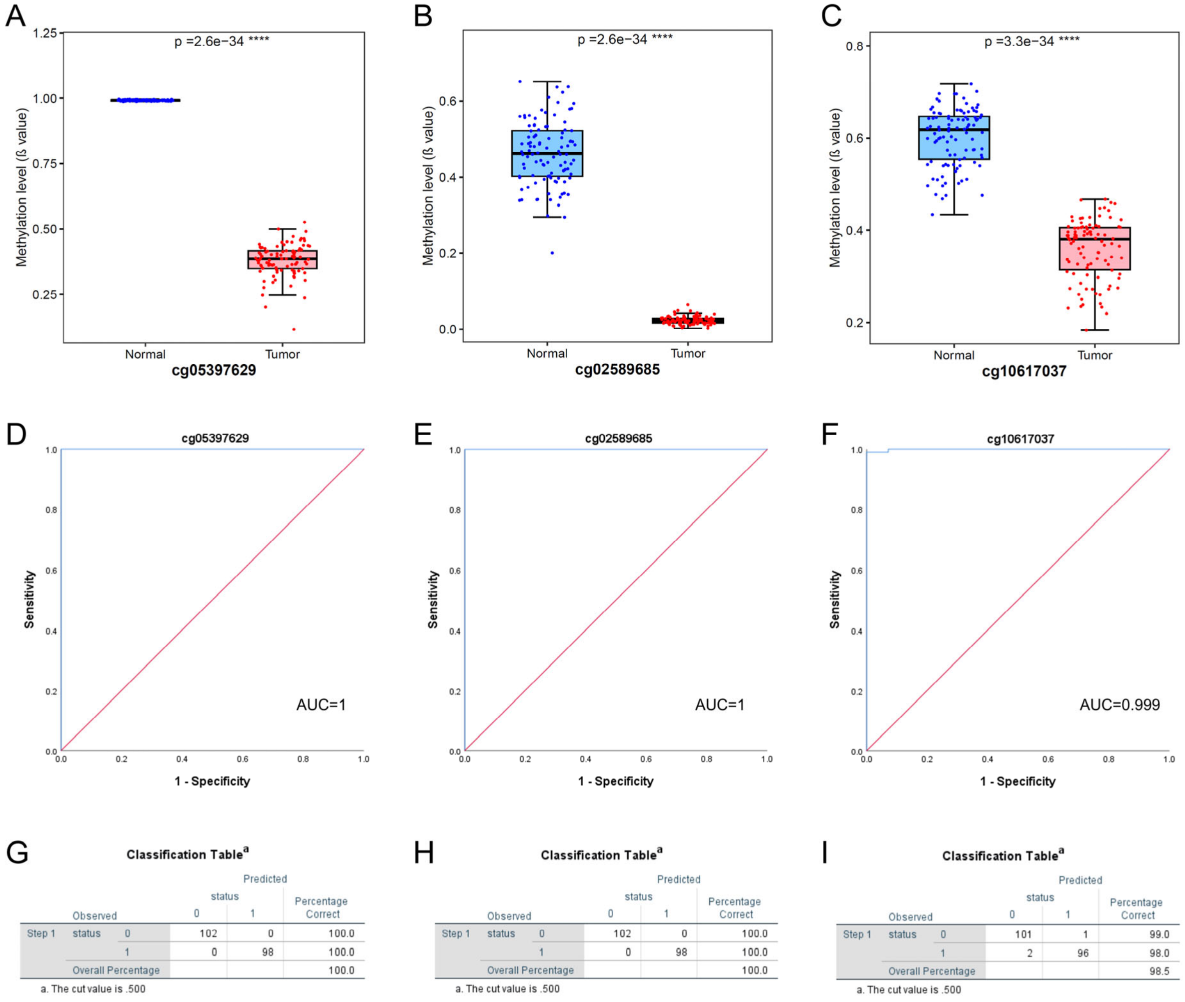


**Figure S4.** Details about distinguishing capacity of three differential methylation probes (DMPs). The *P*-value represents the significance of rank-sum test. ****, <0.0001. The AUC is calculated from the area under the ROC curve. The classification tables were generated from logistic models for CpG and breast cancer. Abbreviations: AUC, area under curve.

**Table S1.** Information of 524 differentially methylation probes (DMPs).

The tables S1 and S2 are uploaded as an additional file (Excel).

**Table S2.** Information of 524 differentially methylation probes (DMPs).

The tables S1 and S2 are uploaded as an additional file (Excel).

**Table S3.** Gene ontology terms that were significantly enriched in an analysis of differentially methylation probes (DMPs).

| | ONTOLOGY | ID | Description | GeneRatio | BgRatio | pvalue | p.adjust | qvalue | geneID | Count |
|---|---|---|---|---|---|---|---|---|---|---|
| GO:0030667 | CC | GO:0030667 | secretory granule membrane | 14/267 | 312/19594 | 0.000104 | 0.041983 | 0.038845 | 391/9342/7879/8904/161/5795/9725/3708/1729/51143/11031/8895/338339/5023 | 14 |

**Table S4.** Gene ontology terms that were significantly enriched in an analysis of differentially methylation regions (DMRs).

| | ONTOLOGY | ID | Description | GeneRatio | BgRatio | pvalue | p.adjust | qvalue | geneID | Count |
|---|---|---|---|---|---|---|---|---|---|---|
| GO:0048568 | BP | GO:0048568 | embryonic organ development | 43/656 | 449/18800 | 2.25E-09 | 1.04E-05 | 8.93E-06 | 3205/ 6299/ 60529/ 5080/ 7545/ 2201/ 3207/ 3481/ 3784/ 7042/ 3204/ 8092/ 11078/ 3249/ 23414/ 5914/ 8854/ 121227/ 2668/ 8323/ 7472/ 4762/ 2290/ 430/ 57545/ 7291/ 1909/ 10653/ 3199/ 56034/ 3217/ 4487/ 4254/ 3236/ 79977/ 639/ 2202/ 220/ 668/ 9096/ 6491/ 585/ 7020 | 43 |
| GO:0097485 | BP | GO:0097485 | neuron projection guidance | 28/656 | 235/18800 | 1.50E-08 | 6.96E-05 | 2.87E-05 | 2044/ 389549/ 5080/ 137970/ 5364/ 53942/ 10570/ 9369/ 54538/ 627/ 6091/ 91624/ 2668/ 10752/ 23768/ 115557/ 4009/ 2290/ 6586/ 1909/ 214/ 3199/ 9378/ 9353/ 399/ 80031/ 9048/ 1630 | 28 |
| GO:0030326 | BP | GO:0030326 | embryonic limb morphogenesis | 19/656 | 119/18800 | 2.98E-08 | 0.000138 | 2.87E-05 | 6423/ 6299/ 60529/ 2201/ 3207/ 340419/ 4488/ 7042/ 8854/ 22943/ 27152/ 8323/ 7291/ 4487/ 3236/ 3238/ 79977/ 6910/ 7020 | 19 |
| GO:0035113 | BP | GO:0035113 | embryonic appendage morphogenesis | 19/656 | 119/18800 | 2.98E-08 | 0.000138 | 2.87E-05 | 6423/ 6299/ 60529/ 2201/ 3207/ 340419/ 4488/ 7042/ 8854/ 22943/ 27152/ 8323/ 7291/ 4487/ 3236/ 3238/ 79977/ 6910/ 7020 | 19 |
| GO:0098742 | BP | GO:0098742 | cell-cell adhesion via plasma-membrane adhesion molecules | 30/656 | 279/18800 | 4.90E-08 | 0.000227 | 2.87E-05 | 56114/ 1825/ 5010/ 1006/ 137970/ 7042/ 56132/ 869/ 56147/ 54538/ 1003/ 1005/ 2895/ 83872/ 6091/ 24146/ 56126/ 91624/ 26011/ 28316/ 22865/ 214/ 57689/ 22871/ 1824/ 140628/ 5100/ 1365/ 56121/ 2195 | 30 |
| GO:0007411 | BP | GO:0007411 | axon guidance | 27/656 | 234/18800 | 5.35E-08 | 0.000248 | 2.87E-05 | 2044/ 389549/ 5080/ 137970/ 5364/ 53942/ 9369/ 54538/ 627/ 6091/ 91624/ 2668/ 10752/ 23768/ 115557/ 4009/ 2290/ 6586/ 1909/ 214/ 3199/ 9378/ 9353/ 399/ 80031/ 9048/ 1630 | 27 |
| GO:0048736 | BP | GO:0048736 | appendage development | 23/656 | 177/18800 | 5.86E-08 | 0.000271 | 2.87E-05 | 6423/ 6299/ 60529/ 2201/ 3207/ 340419/ 3664/ 4488/ 5066/ 7042/ 5914/ 8854/ 22943/ 27152/ 8323/ 3229/ 7291/ 4487/ 3236/ 3238/ 79977/ 6910/ 7020 | 23 |
| GO:0060173 | BP | GO:0060173 | limb development | 23/656 | 177/18800 | 5.86E-08 | 0.000271 | 2.87E-05 | 6423/ 6299/ 60529/ 2201/ 3207/ 340419/ 3664/ 4488/ 5066/ 7042/ 5914/ 8854/ 22943/ 27152/ 8323/ 3229/ 7291/ 4487/ 3236/ 3238/ 79977/ 6910/ 7020 | 23 |
| GO:0060324 | BP | GO:0060324 | face development | 12/656 | 48/18800 | 6.49E-08 | 0.0003 | 2.87E-05 | 84159/ 83690/ 5914/ 8854/ 22943/ 5083/ 1909/ 4487/ 4313/ 79977/ 220/ 585 | 12 |
| GO:0090596 | BP | GO:0090596 | sensory organ morphogenesis | 28/656 | 266/18800 | 2.14E-07 | 0.000989 | 8.50E-05 | 7068/ 5080/ 7545/ 2201/ 3784/ 11078/ 3249/ 4211/ 121227/ 27152/ 8323/ 7472/ 4762/ 2290/ 3229/ 6015/ 7291/ 1909/ 3199/ 4487/ 639/ 2202/ 220/ 668/ 9096/ 2195/ 585/ 7020 | 28 |
| GO:0016331 | BP | GO:0016331 | morphogenesis of embryonic epithelium | 20/656 | 150/18800 | 2.79E-07 | 0.001292 | 0.000101 | 6423/ 6046/ 7042/ 5914/ 8854/ 2668/ 8323/ 7472/ 57545/ 7291/ 10653/ 79977/ 5100/ 10395/ 220/ 9096/ 6491/ 585/ 7041/ 7020 | 20 |
| GO:0048705 | BP | GO:0048705 | skeletal system morphogenesis | 25/656 | 228/18800 | 4.42E-07 | 0.002046 | 0.00013 | 6423/ 3205/ 60529/ 2201/ 3207/ 4488/ 84159/ 2268/ 2690/ 3204/ 8092/ 79925/ 5914/ 4762/ 4057/ 7291/ 3199/ 55790/ 3217/ 4487/ 3236/ 4313/ 79977/ 134701/ 7020 | 25 |

| | | | | | | | | | | |
|---|---|---|---|---|---|---|---|---|---|---|
| GO:0035136 | BP | GO:0035136 | forelimb morphogenesis | 10/656 | 38/18800 | 4.88E-07 | 0.002258 | 0.00013 | 60529/ 3207/ 340419/ 4488/ 8854/ 7291/ 4487/ 3236/ 6910/ 7020 | 10 |
| GO:0048562 | BP | GO:0048562 | embryonic organ morphogenesis | 29/656 | 294/18800 | 5.15E-07 | 0.002382 | 0.00013 | 3205/ 60529/ 5080/ 7545/ 2201/ 3207/ 3784/ 3204/ 8092/ 11078/ 3249/ 121227/ 8323/ 4762/ 2290/ 7291/ 1909/ 3199/ 3217/ 4487/ 3236/ 79977/ 2202/ 220/ 668/ 9096/ 6491/ 585/ 7020 | 29 |
| GO:0035107 | BP | GO:0035107 | appendage morphogenesis | 19/656 | 142/18800 | 5.23E-07 | 0.002419 | 0.00013 | 6423/ 6299/ 60529/ 2201/ 3207/ 340419/ 4488/ 7042/ 8854/ 22943/ 27152/ 8323/ 7291/ 4487/ 3236/ 3238/ 79977/ 6910/ 7020 | 19 |
| GO:0035108 | BP | GO:0035108 | limb morphogenesis | 19/656 | 142/18800 | 5.23E-07 | 0.002419 | 0.00013 | 6423/ 6299/ 60529/ 2201/ 3207/ 340419/ 4488/ 7042/ 8854/ 22943/ 27152/ 8323/ 7291/ 4487/ 3236/ 3238/ 79977/ 6910/ 7020 | 19 |
| GO:0060078 | BP | GO:0060078 | regulation of postsynaptic membrane potential | 18/656 | 131/18800 | 6.98E-07 | 0.003228 | 0.000162 | 6622/ 2567/ 56853/ 2898/ 1140/ 869/ 23316/ 2895/ 2555/ 2897/ 401190/ 18/ 2559/ 2911/ 22871/ 9378/ 2557/ 2030 | 18 |
| GO:0035115 | BP | GO:0035115 | embryonic forelimb morphogenesis | 9/656 | 31/18800 | 7.35E-07 | 0.003401 | 0.000162 | 60529/ 3207/ 340419/ 4488/ 8854/ 7291/ 4487/ 6910/ 7020 | 9 |
| GO:0007389 | BP | GO:0007389 | pattern specification process | 38/656 | 463/18800 | 1.02E-06 | 0.004743 | 0.000204 | 6423/ 3205/ 389549/ 7092/ 60529/ 5080/ 7545/ 3207/ 4488/ 2066/ 3204/ 6091/ 4211/ 8854/ 22943/ 27152/ 2668/ 7472/ 4762/ 2290/ 3229/ 57545/ 6015/ 1745/ 3199/ 3217/ 10655/ 4487/ 3236/ 92749/ 3238/ 134701/ 140628/ 5100/ 6910/ 9096/ 6491/ 585 | 38 |
| GO:0045664 | BP | GO:0045664 | regulation of neuron differentiation | 22/656 | 192/18800 | 1.03E-06 | 0.004747 | 0.000204 | 6423/ 1910/ 389549/ 5080/ 57795/ 627/ 4211/ 5914/ 22943/ 4762/ 4009/ 2290/ 7101/ 4803/ 238/ 1745/ 3199/ 22871/ 30012/ 4058/ 4825/ 79727 | 22 |
| GO:0051932 | BP | GO:0051932 | synaptic transmission, GABAergic | 11/656 | 51/18800 | 1.15E-06 | 0.005306 | 0.000217 | 2567/ 2555/ 6869/ 8224/ 6529/ 2559/ 22865/ 22871/ 2557/ 766/ 148 | 11 |
| GO:0061564 | BP | GO:0061564 | axon development | 38/656 | 479/18800 | 2.32E-06 | 0.010754 | 0.00042 | 2044/ 389549/ 5080/ 2917/ 1496/ 137970/ 8578/ 5364/ 53942/ 9369/ 54538/ 627/ 6091/ 4929/ 91624/ 2668/ 10752/ 23768/ 115557/ 4009/ 2290/ 7101/ 4803/ 6586/ 22865/ 1909/ 214/ 3199/ 57689/ 9378/ 4313/ 9353/ 399/ 80031/ 9048/ 1630/ 4825/ 706 | 38 |
| GO:0042391 | BP | GO:0042391 | regulation of membrane potential | 35/656 | 425/18800 | 2.54E-06 | 0.011741 | 0.000439 | 6622/ 2567/ 56853/ 3781/ 2898/ 1140/ 3784/ 783/ 869/ 23316/ 2895/ 63895/ 2555/ 40/ 2897/ 401190/ 10008/ 3775/ 6869/ 18/ 2559/ 2911/ 22871/ 3728/ 9378/ 1824/ 2557/ 6910/ 54207/ 2030/ 706/ 64208/ 50801/ 9096/ 148 | 35 |
| GO:0042733 | BP | GO:0042733 | embryonic digit morphogenesis | 11/656 | 58/18800 | 4.39E-06 | 0.020321 | 0.000725 | 6423/ 6299/ 60529/ 3207/ 4488/ 27152/ 8323/ 7291/ 4487/ 3238/ 79977 | 11 |
| GO:0008406 | BP | GO:0008406 | gonad development | 23/656 | 226/18800 | 4.56E-06 | 0.021105 | 0.000725 | 6423/ 6299/ 3207/ 2674/ 2099/ 84159/ 9985/ 7042/ 5872/ 1299/ 5806/ 23414/ 5914/ 5798/ 11086/ 6586/ 4254/ 132625/ 4313/ 9353/ 23626/ 675/ 668 | 23 |
| GO:1903522 | BP | GO:1903522 | regulation of blood circulation | 25/656 | 260/18800 | 4.92E-06 | 0.022769 | 0.000752 | 7068/ 1910/ 3781/ 10060/ 3784/ 7042/ 1129/ 783/ 5142/ 40/ 10008/ 5020/ 5144/ 6869/ 2166/ 1909/ 3728/ 4313/ 1824/ 5743/ 6910/ 6870/ 3350/ 9096/ 148 | 25 |

| | | | | | | | | | | |
|---|---|---|---|---|---|---|---|---|---|---|
| GO:0034329 | BP | GO:0034329 | cell junction assembly | 34/656 | 420/18800 | 5.12E-06 | 0.023697 | 0.00752 | 6622/ 2066/ 5010/ 1006/ 153562/ 5364/ 5872/ 53942/ 56132/ 869/ 627/ 1003/ 23316/ 1005/ 2895/ 2555/ 24146/ 91862/ 56126/ 40/ 22943/ 28316/ 23768/ 5020/ 79953/ 22865/ 22871/ 3728/ 9378/ 79977/ 6910/ 10395/ 23136/ 1365 | 34 |
| GO:0030534 | BP | GO:0030534 | adult behavior | 17/656 | 136/18800 | 5.30E-06 | 0.024524 | 0.00752 | 4852/ 6622/ 7545/ 1140/ 9369/ 80243/ 4929/ 2668/ 10752/ 285671/ 18/ 7200/ 238/ 9378/ 3236/ 8772/ 585 | 17 |
| GO:0051960 | BP | GO:0051960 | regulation of nervous system development | 35/656 | 440/18800 | 5.49E-06 | 0.025395 | 0.00752 | 389549/ 4311/ 4049/ 5080/ 5364/ 869/ 627/ 23316/ 2895/ 6091/ 400746/ 5914/ 40/ 22943/ 26011/ 84504/ 6258/ 23768/ 5020/ 7472/ 79953/ 2290/ 7101/ 430/ 4803/ 1745/ 22865/ 22871/ 9378/ 9353/ 80031/ 1630/ 4825/ 706/ 79727 | 35 |
| GO:0009952 | BP | GO:0009952 | anterior/posterior pattern specification | 22/656 | 214/18800 | 6.22E-06 | 0.028773 | 0.00824 | 6423/ 3205/ 389549/ 60529/ 5080/ 3207/ 4488/ 3204/ 8854/ 22943/ 7472/ 4762/ 3229/ 6015/ 3199/ 3217/ 10655/ 4487/ 3236/ 134701/ 5100/ 9096 | 22 |
| GO:0045137 | BP | GO:0045137 | development of primary sexual characteristics | 23/656 | 231/18800 | 6.58E-06 | 0.030432 | 0.00844 | 6423/ 6299/ 3207/ 2674/ 2099/ 84159/ 9985/ 7042/ 5872/ 1299/ 5806/ 23414/ 5914/ 5798/ 11086/ 6586/ 4254/ 132625/ 4313/ 9353/ 23626/ 675/ 668 | 23 |
| GO:0021953 | BP | GO:0021953 | central nervous system neuron differentiation | 19/656 | 169/18800 | 7.32E-06 | 0.033861 | 0.00909 | 26468/ 4852/ 5080/ 84871/ 161357/ 869/ 219409/ 2895/ 4929/ 401/ 1996/ 4009/ 2290/ 7101/ 1745/ 3236/ 9353/ 1630/ 4825 | 19 |
| GO:0007409 | BP | GO:0007409 | axonogenesis | 34/656 | 430/18800 | 8.48E-06 | 0.039239 | 0.01022 | 2044/ 389549/ 5080/ 1496/ 137970/ 5364/ 53942/ 9369/ 54538/ 627/ 6091/ 4929/ 91624/ 2668/ 10752/ 23768/ 115557/ 4009/ 2290/ 7101/ 4803/ 6586/ 22865/ 1909/ 214/ 3199/ 57689/ 9378/ 9353/ 399/ 80031/ 9048/ 1630/ 4825 | 34 |
| GO:0048706 | BP | GO:0048706 | embryonic skeletal system development | 16/656 | 127/18800 | 9.01E-06 | 0.041695 | 0.01054 | 3205/ 60529/ 3207/ 3204/ 8092/ 3231/ 56243/ 1745/ 7291/ 1909/ 3199/ 3217/ 10655/ 3236/ 79977/ 7020 | 16 |
| GO:0048608 | BP | GO:0048608 | reproductive structure development | 34/656 | 433/18800 | 9.83E-06 | 0.045471 | 0.01116 | 6423/ 4311/ 6299/ 3207/ 2674/ 2099/ 84159/ 9985/ 3481/ 7042/ 5872/ 1299/ 5806/ 23414/ 5914/ 9420/ 7472/ 5798/ 4762/ 430/ 11086/ 6586/ 10653/ 4254/ 132625/ 4313/ 9353/ 23626/ 5743/ 79977/ 639/ 675/ 668/ 51673 | 34 |
| GO:0051965 | BP | GO:0051965 | positive regulation of synapse assembly | 11/656 | 63/18800 | 1.01E-05 | 0.046913 | 0.0112 | 869/ 627/ 23316/ 2895/ 40/ 23768/ 5020/ 79953/ 22865/ 22871/ 9378 | 11 |
| GO:0098793 | CC | GO:0098793 | presynapse | 42/694 | 492/19594 | 1.44E-07 | 6.95E-05 | 6.28E-05 | 4852/ 5179/ 4311/ 6622/ 2917/ 93664/ 2898/ 2066/ 9892/ 6456/ 143425/ 1006/ 114569/ 9829/ 5872/ 6530/ 53942/ 91752/ 783/ 6857/ 6854/ 627/ 5142/ 8618/ 2555/ 6539/ 401190/ 5020/ 5798/ 79953/ 8224/ 6529/ 80863/ 4803/ 284612/ 148281/ 22871/ 9378/ 22895/ 5100/ 2030/ 148 | 42 |
| GO:0097060 | CC | GO:0097060 | synaptic membrane | 34/694 | 373/19594 | 5.07E-07 | 0.00244 | 0.0011 | 2567/ 2917/ 2898/ 2066/ 1140/ 9892/ 2893/ 1006/ 9123/ 1129/ 6530/ 53942/ 6857/ 869/ 2895/ 2555/ 2897/ 401190/ 79953/ 3775/ 6529/ 80863/ 2559/ 22865/ 57689/ 2911/ 22871/ 9378/ 22895/ 5100/ 2557/ 399694/ 1630/ 148 | 34 |
| GO:0045211 | CC | GO:0045211 | postsynaptic membrane | 27/694 | 271/19594 | 1.40E-06 | 0.00674 | 0.00203 | 2567/ 2917/ 2898/ 2066/ 1140/ 2893/ 9123/ 1129/ 869/ 2895/ 2555/ 2897/ 401190/ 79953/ 6529/ 80863/ 2559/ 22865/ 57689/ 2911/ 22871/ 22895/ 5100/ 2557/ 399694/ 1630/ 148 | 27 |
| GO:0070382 | CC | GO:0070382 | exocytic vesicle | 21/694 | 214/19594 | 2.56E-05 | 0.012329 | 0.02786 | 5179/ 4311/ 6622/ 9892/ 143425/ 114569/ 5872/ 6857/ 6854/ 627/ 5142/ 2555/ 79953/ 8224/ 80863/ 4803/ 284612/ 148281/ 22895/ 1809/ 84258 | 21 |

| | | | | | | | | | | |
|---|---|---|---|---|---|---|---|---|---|---|
| GO:0043025 | CC | GO:0043025 | neuronal cell body | 35/694 | 482/19594 | 5.20E-05 | 0.025004 | 0.004477 | 2044/ 4852/ 5179/ 4311/ 6622/ 2674/ 3781/ 2898/ 5066/ 2893/ 57795/ 80333/ 5137/ 3784/ 7042/ 6530/ 2690/ 91752/ 3249/ 2555/ 8470/ 40/ 5153/ 401190/ 10008/ 5798/ 3775/ 1996/ 4762/ 6529/ 214/ 9378/ 6870/ 5126/ 900 | 35 |
| GO:0099634 | CC | GO:0099634 | postsynaptic specialization membrane | 14/694 | 116/19594 | 6.18E-05 | 0.029719 | 0.004477 | 2066/ 1140/ 9123/ 2895/ 2555/ 401190/ 79953/ 80863/ 22865/ 57689/ 2911/ 22871/ 2557/ 1630 | 14 |
| GO:0080021 | CC | GO:0080021 | synaptic vesicle | 19/694 | 196/19594 | 7.24E-05 | 0.034844 | 0.004499 | 5179/ 4311/ 6622/ 9892/ 143425/ 114569/ 5872/ 6857/ 6854/ 627/ 5142/ 2555/ 79953/ 8224/ 80863/ 4803/ 284612/ 148281/ 22895 | 19 |
| GO:0008503 | MF | GO:0008503 | benzodiazepine receptor activity | 5/672 | 11/18410 | 2.46E-05 | 0.019428 | 0.017451 | 2567/ 2555/ 2559/ 2557/ 706 | 5 |
| GO:0030594 | MF | GO:0030594 | neurotransmitter receptor activity | 14/672 | 111/18410 | 5.21E-05 | 0.041174 | 0.018493 | 2567/ 2898/ 1140/ 2893/ 1129/ 2895/ 2555/ 2897/ 2559/ 2911/ 3354/ 2557/ 3350/ 706 | 14 |

**Table S5.** Correlation analysis between plasma one-carbon metabolism-related micronutrients and 14 differential methylation probes (DMPs).

| Micronutrients | Probes | r | Crude *P*-value | FDR *P*-value | Bonferroni *P*-value |
|---|---|---|---|---|---|
| Methionine | cg15740243 | 0.186342 | 0.008243 | 0.017517 | 0.140138 |
| | cg21042336 | 0.106159 | 0.134618 | 0.134618 | 1 |
| | cg04622888 | 0.136532 | 0.053881 | 0.064311 | 0.915985 |
| | cg00901687 | 0.132963 | 0.060528 | 0.064311 | 1 |
| | cg13824270 | -0.23717 | 0.000721 | 0.003494 | **0.01226** |
| | cg03363289 | 0.165831 | 0.018937 | 0.029266 | 0.321923 |
| | cg04794268 | 0.226955 | 0.00123 | 0.004183 | **0.020913** |
| | cg24312537 | 0.133792 | 0.058926 | 0.064311 | 1 |
| | cg21697381 | 0.234701 | 0.000822 | 0.003494 | **0.013977** |
| | cg01701207 | 0.150085 | 0.033901 | 0.044332 | 0.576314 |
| | cg09491380 | 0.167952 | 0.017443 | 0.029266 | 0.296535 |
| | cg04657470 | 0.193628 | 0.006011 | 0.014597 | 0.102182 |
| | cg25755428 | -0.18302 | 0.009486 | 0.017917 | 0.161254 |
| | cg06536614 | 0.158763 | 0.02474 | 0.035048 | 0.420581 |
| Cysteine | cg15740243 | -0.19126 | 0.006667 | 0.014168 | 0.113344 |
| | cg21042336 | -0.22144 | 0.001626 | 0.007503 | **0.027638** |
| | cg04622888 | -0.05351 | 0.451699 | 0.47993 | 1 |
| | cg00901687 | -0.10239 | 0.149094 | 0.194969 | 1 |
| | cg13824270 | 0.14778 | 0.03677 | 0.052091 | 0.625097 |
| | cg03363289 | -0.0871 | 0.22007 | 0.249413 | 1 |
| | cg04794268 | -0.181 | 0.010322 | 0.019497 | 0.175471 |
| | cg24312537 | -0.22182 | 0.001595 | 0.007503 | **0.027122** |
| | cg21697381 | -0.09719 | 0.170962 | 0.207597 | 1 |
| | cg01701207 | -0.21979 | 0.001765 | 0.007503 | **0.030013** |
| | cg09491380 | -0.20395 | 0.00377 | 0.011367 | 0.064092 |
| | cg04657470 | -0.17722 | 0.012059 | 0.0205 | 0.204997 |
| | cg25755428 | 0.014576 | 0.837691 | 0.837691 | 1 |
| | cg06536614 | -0.15123 | 0.032548 | 0.050302 | 0.553318 |
| Homocysteine | cg15740243 | 0.008995 | 0.8994 | 0.955613 | 1 |
| | cg21042336 | 0.048811 | 0.492475 | 0.909338 | 1 |
| | cg04622888 | 0.014693 | 0.836405 | 0.947926 | 1 |
| | cg00901687 | 0.095656 | 0.177854 | 0.909338 | 1 |
| | cg13824270 | -0.05724 | 0.42075 | 0.909338 | 1 |
| | cg03363289 | 0.002599 | 0.970862 | 0.970862 | 1 |
| | cg04794268 | 0.047965 | 0.500014 | 0.909338 | 1 |
| | cg24312537 | 0.026797 | 0.70643 | 0.909338 | 1 |
| | cg21697381 | 0.103145 | 0.146107 | 0.909338 | 1 |
| | cg01701207 | 0.043544 | 0.54038 | 0.909338 | 1 |
| | cg09491380 | 0.04369 | 0.539021 | 0.909338 | 1 |
| | cg04657470 | 0.025005 | 0.725243 | 0.909338 | 1 |

| | | | | | |
|---|---|---|---|---|---|
| | cg25755428 | 0.038743 | 0.585971 | 0.909338 | 1 |
| | cg06536614 | -0.04232 | 0.551816 | 0.909338 | 1 |
| SAM | cg15740243 | 0.274309 | 8.47E-05 | 0.00018 | **0.00144** |
| | cg21042336 | 0.256091 | 0.000252 | 0.000473 | **0.004284** |
| | cg04622888 | 0.252623 | 0.000307 | 0.000475 | **0.005226** |
| | cg00901687 | 0.224367 | 0.001403 | 0.001491 | **0.023857** |
| | cg13824270 | -0.23941 | 0.000639 | 0.000903 | **0.010867** |
| | cg03363289 | 0.33137 | 1.64E-06 | 5.59E-06 | **2.79E-05** |
| | cg04794268 | 0.383957 | 1.99E-08 | 1.13E-07 | **3.39E-07** |
| | cg24312537 | 0.254382 | 0.000278 | 0.000473 | **0.004727** |
| | cg21697381 | 0.23797 | 0.000691 | 0.000903 | **0.011744** |
| | cg01701207 | 0.22536 | 0.001334 | 0.001491 | **0.022686** |
| | cg09491380 | 0.291122 | 2.89E-05 | 7.01E-05 | **0.000491** |
| | cg04657470 | 0.322525 | 3.20E-06 | 9.06E-06 | **5.44E-05** |
| | cg25755428 | -0.19338 | 0.006077 | 0.006077 | 0.103312 |
| | cg06536614 | 0.229493 | 0.00108 | 0.001311 | **0.018353** |
| SAH | cg15740243 | 0.071905 | 0.311624 | 0.4532 | 1 |
| | cg21042336 | 0.152975 | 0.030574 | 0.176197 | 0.51976 |
| | cg04622888 | 0.022217 | 0.754842 | 0.754842 | 1 |
| | cg00901687 | 0.100723 | 0.155863 | 0.4532 | 1 |
| | cg13824270 | -0.04275 | 0.547776 | 0.582012 | 1 |
| | cg03363289 | 0.069886 | 0.325436 | 0.4532 | 1 |
| | cg04794268 | 0.081579 | 0.25081 | 0.4532 | 1 |
| | cg24312537 | 0.062192 | 0.381646 | 0.4532 | 1 |
| | cg21697381 | 0.086587 | 0.222793 | 0.4532 | 1 |
| | cg01701207 | 0.059848 | 0.399882 | 0.4532 | 1 |
| | cg09491380 | 0.085573 | 0.228278 | 0.4532 | 1 |
| | cg04657470 | 0.078502 | 0.269188 | 0.4532 | 1 |
| | cg25755428 | -0.15251 | 0.031094 | 0.176197 | 0.528592 |
| | cg06536614 | 0.167721 | 0.017601 | 0.176197 | 0.299213 |
| Folate | cg15740243 | 0.335299 | 1.21E-06 | 3.39E-06 | **2.06E-05** |
| | cg21042336 | 0.274624 | 8.31E-05 | 0.000128 | **0.001412** |
| | cg04622888 | 0.165134 | 0.019451 | 0.022045 | 0.330671 |
| | cg00901687 | 0.144839 | 0.040728 | 0.043273 | 0.692371 |
| | cg13824270 | -0.16847 | 0.017096 | 0.02076 | 0.290638 |
| | cg03363289 | 0.238393 | 0.000675 | 0.000957 | **0.011481** |
| | cg04794268 | 0.333493 | 1.40E-06 | 3.39E-06 | **2.37E-05** |
| | cg24312537 | 0.329249 | 1.93E-06 | 4.10E-06 | **3.28E-05** |
| | cg21697381 | 0.323377 | 3.00E-06 | 5.67E-06 | **5.10E-05** |
| | cg01701207 | 0.33619 | 1.13E-06 | 3.39E-06 | **1.93E-05** |
| | cg09491380 | 0.360896 | 1.52E-07 | 8.63E-07 | **2.59E-06** |
| | cg04657470 | 0.289215 | 3.27E-05 | 5.56E-05 | **0.000556** |
| | cg25755428 | -0.12583 | 0.075822 | 0.075822 | 1 |
| | cg06536614 | 0.231087 | 0.000994 | 0.0013 | **0.016896** |
| 5-MTHF | cg15740243 | -0.00653 | 0.926867 | 0.967454 | 1 |

| | | | | | |
|---|---|---|---|---|---|
| | cg21042336 | 0.025796 | 0.716917 | 0.967454 | 1 |
| | cg04622888 | 0.01993 | 0.77939 | 0.967454 | 1 |
| | cg00901687 | -0.1941 | 0.005886 | 0.100069 | 0.100069 |
| | cg13824270 | 0.07655 | 0.28131 | 0.613435 | 1 |
| | cg03363289 | -0.0029 | 0.967454 | 0.967454 | 1 |
| | cg04794268 | -0.10069 | 0.156006 | 0.587114 | 1 |
| | cg24312537 | -0.05773 | 0.416803 | 0.708566 | 1 |
| | cg21697381 | -0.00802 | 0.910254 | 0.967454 | 1 |
| | cg01701207 | -0.07539 | 0.288675 | 0.613435 | 1 |
| | cg09491380 | -0.05863 | 0.409544 | 0.708566 | 1 |
| | cg04657470 | -0.01496 | 0.833467 | 0.967454 | 1 |
| | cg25755428 | 0.117377 | 0.097867 | 0.554578 | 1 |
| | cg06536614 | -0.08957 | 0.207217 | 0.587114 | 1 |
| Choline | cg15740243 | 0.179564 | 0.010953 | 0.046548 | 0.186193 |
| | cg21042336 | 0.030174 | 0.671457 | 0.671457 | 1 |
| | cg04622888 | 0.122217 | 0.0847 | 0.110761 | 1 |
| | cg00901687 | 0.04983 | 0.483475 | 0.513693 | 1 |
| | cg13824270 | -0.13399 | 0.058556 | 0.082955 | 0.995459 |
| | cg03363289 | 0.166356 | 0.018557 | 0.047884 | 0.315472 |
| | cg04794268 | 0.149952 | 0.034061 | 0.057904 | 0.579038 |
| | cg24312537 | 0.13618 | 0.054509 | 0.082955 | 0.926648 |
| | cg21697381 | 0.24512 | 0.000468 | 0.007959 | **0.007959** |
| | cg01701207 | 0.107643 | 0.129223 | 0.146453 | 1 |
| | cg09491380 | 0.161262 | 0.022534 | 0.047884 | 0.383073 |
| | cg04657470 | 0.163487 | 0.020715 | 0.047884 | 0.352152 |
| | cg25755428 | -0.16462 | 0.019836 | 0.047884 | 0.33721 |
| | cg06536614 | 0.110165 | 0.12044 | 0.146249 | 1 |
| Betaine | cg15740243 | 0.230258 | 0.001038 | 0.001877 | **0.01764** |
| | cg21042336 | 0.142234 | 0.044526 | 0.047309 | 0.756939 |
| | cg04622888 | 0.242997 | 0.000526 | 0.001118 | **0.008945** |
| | cg00901687 | 0.14704 | 0.037734 | 0.042765 | 0.64147 |
| | cg13824270 | -0.21147 | 0.002648 | 0.003751 | **0.045011** |
| | cg03363289 | 0.293931 | 2.40E-05 | 9.78E-05 | **0.000407** |
| | cg04794268 | 0.297566 | 1.88E-05 | 9.78E-05 | **0.000319** |
| | cg24312537 | 0.211699 | 0.002619 | 0.003751 | **0.044515** |
| | cg21697381 | 0.229057 | 0.001104 | 0.001877 | **0.018772** |
| | cg01701207 | 0.198812 | 0.004769 | 0.006237 | 0.081076 |
| | cg09491380 | 0.248464 | 0.000389 | 0.000944 | **0.006609** |
| | cg04657470 | 0.254304 | 0.000279 | 0.000791 | **0.004748** |
| | cg25755428 | -0.10754 | 0.129589 | 0.129589 | 1 |
| | cg06536614 | 0.162061 | 0.021866 | 0.026551 | 0.371714 |
| P5P | cg15740243 | 0.420745 | 5.53E-10 | 1.57E-09 | **9.40E-09** |
| | cg21042336 | 0.328727 | 2.01E-06 | 2.44E-06 | **3.42E-05** |
| | cg04622888 | 0.2959 | 2.10E-05 | 2.38E-05 | **0.000357** |
| | cg00901687 | 0.375972 | 4.10E-08 | 7.75E-08 | **6.98E-07** |

| | | | | | |
|---|---|---|---|---|---|
| | cg13824270 | -0.36748 | 8.66E-08 | 1.47E-07 | **1.47E-06** |
| | cg03363289 | 0.355233 | 2.45E-07 | 3.78E-07 | **4.16E-06** |
| | cg04794268 | 0.434266 | 1.32E-10 | 5.62E-10 | **2.25E-09** |
| | cg24312537 | 0.404591 | 2.82E-09 | 6.61E-09 | **4.79E-08** |
| | cg21697381 | 0.350242 | 3.69E-07 | 5.23E-07 | **6.28E-06** |
| | cg01701207 | 0.403582 | 3.11E-09 | 6.61E-09 | **5.29E-08** |
| | cg09491380 | 0.424814 | 3.62E-10 | 1.23E-09 | **6.15E-09** |
| | cg04657470 | 0.33324 | 1.42E-06 | 1.86E-06 | **2.42E-05** |
| | cg25755428 | -0.20277 | 0.003982 | 0.004231 | 0.067689 |
| | cg06536614 | 0.126829 | 0.073514 | 0.073514 | 1 |
| Vitamin B2 | cg15740243 | 0.389228 | 1.22E-08 | 4.16E-08 | **2.08E-07** |
| | cg21042336 | 0.293039 | 2.54E-05 | 3.60E-05 | **0.000432** |
| | cg04622888 | 0.291379 | 2.84E-05 | 3.71E-05 | **0.000482** |
| | cg00901687 | 0.26825 | 0.000123 | 0.000138 | **0.002087** |
| | cg13824270 | -0.33273 | 1.48E-06 | 2.80E-06 | **2.52E-05** |
| | cg03363289 | 0.363037 | 1.27E-07 | 3.55E-07 | **2.16E-06** |
| | cg04794268 | 0.40846 | 1.92E-09 | 1.09E-08 | **3.27E-08** |
| | cg24312537 | 0.31714 | 4.75E-06 | 8.07E-06 | **8.07E-05** |
| | cg21697381 | 0.348994 | 4.09E-07 | 8.69E-07 | **6.95E-06** |
| | cg01701207 | 0.300679 | 1.52E-05 | 2.35E-05 | **0.000258** |
| | cg09491380 | 0.361362 | 1.46E-07 | 3.55E-07 | **2.49E-06** |
| | cg04657470 | 0.283423 | 4.77E-05 | 5.79E-05 | **0.00081** |
| | cg25755428 | -0.26734 | 0.00013 | 0.000138 | **0.002206** |
| | cg06536614 | 0.214254 | 0.002315 | 0.002315 | **0.039362** |
| Vitamin B12 | cg15740243 | 0.337764 | 1.00E-06 | 2.13E-06 | **1.70E-05** |
| | cg21042336 | 0.307775 | 9.28E-06 | 1.75E-05 | **0.000158** |
| | cg04622888 | 0.304148 | 1.19E-05 | 2.03E-05 | **0.000203** |
| | cg00901687 | 0.284372 | 4.48E-05 | 5.86E-05 | **0.000762** |
| | cg13824270 | -0.26706 | 0.000132 | 0.00015 | **0.002243** |
| | cg03363289 | 0.381196 | 2.56E-08 | 8.72E-08 | **4.36E-07** |
| | cg04794268 | 0.402901 | 3.32E-09 | 1.41E-08 | **5.65E-08** |
| | cg24312537 | 0.294662 | 2.28E-05 | 3.23E-05 | **0.000388** |
| | cg21697381 | 0.366565 | 9.37E-08 | 2.66E-07 | **1.59E-06** |
| | cg01701207 | 0.300808 | 1.50E-05 | 2.32E-05 | **0.000256** |
| | cg09491380 | 0.349637 | 3.88E-07 | 9.43E-07 | **6.60E-06** |
| | cg04657470 | 0.276029 | 7.61E-05 | 9.24E-05 | **0.001294** |
| | cg25755428 | -0.1095 | 0.122725 | 0.122725 | 1 |
| | cg06536614 | 0.127003 | 0.073118 | 0.077688 | 1 |

Abbreviations: FDR, false discovery rate; P5P, pyridoxal 5-phosphate; SAM, S-adenosylmethionine; SAH, S-adenosylhomocysteine.

**Table S6.** Mediation analysis of differential methylation probes (DMPs) in the association between plasma one-carbon metabolism-related nutrients and risk of breast cancer.

| Micronutrients | CpG | R | Total effect β | Direct effect β | Indirect effect β | Proportion |
|---|---|---|---|---|---|---|
| Methionine | cg13824270 | -0.237* | -0.0862*** | -0.0353 | -0.0510*** | **60.35%*** ** |
| | cg04794268 | 0.236* | -0.1265*** | -0.0366 | -0.0899*** | **72.88%*** ** |
| | cg21697381 | 0.221* | -0.0796 | -0.0324 | -0.0472 | 62.31% |
| Cysteine | cg21042336 | -0.221* | 0.0493 | 0.0165 | 0.0327* | 50.17% |
| | cg24312537 | -0.222* | 0.0716* | 0.0375 | 0.0341 | 47.99% |
| | cg01701207 | -0.220* | 0.0621 | 0.0264 | 0.0356 | 55.64% |
| SAM | cg15740243 | 0.274** | -0.2694*** | -0.1843*** | -0.0851*** | **32.06%*** ** |
| | cg21042336 | 0.256** | -0.2369*** | -0.2121*** | -0.0248 | 10.81% |
| | cg04622888 | 0.253** | -0.2111*** | -0.1680*** | -0.0431* | **21.20%* ** |
| | cg00901687 | 0.224* | -0.2392*** | -0.2185 | -0.0207 | 9.01% |
| | cg13824270 | -0.239* | -0.2307*** | -0.2004*** | -0.0303*** | **13.13%*** ** |
| | cg03363289 | 0.331*** | -0.2043*** | -0.1516*** | -0.0527*** | **26.43%*** ** |
| | cg04794268 | 0.384*** | -0.2610*** | -0.129 | -0.1320*** | **51.20%*** ** |
| | cg24312537 | 0.254*** | -0.3046*** | -0.2295*** | -0.0751*** | **24.61%*** ** |
| | cg21697381 | 0.238* | -0.2162*** | -0.1442*** | -0.0720*** | **33.35%*** ** |
| | cg01701207 | 0.225* | -0.3221*** | -0.2602*** | -0.0619*** | **18.99%*** ** |
| | cg09491380 | 0.291*** | -0.2925*** | -0.2035*** | -0.0889*** | **29.93%*** ** |
| | cg04657470 | 0.323*** | -0.2214*** | -0.1722* | -0.0493*** | **22.22%*** ** |
| | cg06536614 | 0.229* | -0.2551*** | -0.2376*** | -0.0175* | **6.86%* ** |
| Folate | cg15740243 | 0.335*** | -0.1184* | -0.0468 | -0.0716*** | **63.41%* ** |
| | cg21042336 | 0.275** | -0.1083*** | -0.0534 | -0.0549*** | **51.77%*** ** |
| | cg03363289 | 0.238* | -0.1670*** | -0.1240*** | -0.0430 | 26.90% |
| | cg04794268 | 0.333*** | -0.1690*** | -0.0361 | -0.1328*** | **79.43%*** ** |
| | cg24312537 | 0.329*** | -0.1288* | -0.0697* | -0.0591*** | **44.26%* ** |
| | cg21697381 | 0.323*** | -0.0908* | -0.0249 | -0.0659* | **75.80%*** ** |
| | cg01701207 | 0.336*** | -0.1343*** | -0.0725* | -0.0619*** | **47.34%*** ** |
| | cg09491380 | 0.361*** | -0.1230* | -0.0495 | -0.0734*** | **62.48%* ** |
| | cg04657470 | 0.289*** | -0.1312*** | -0.0890*** | -0.0423 | 33.75% |
| | cg06536614 | 0.231* | -0.1512*** | -0.1236*** | -0.0275* | **18.95%* ** |
| Choline | cg21697381 | 0.245** | -0.1014* | -0.0309 | -0.0705* | **71.49%*** ** |
| Betaine | cg15740243 | 0.230* | -0.1442* | -0.0904 | -0.0538*** | **33.22%* ** |
| | cg04622888 | 0.243** | -0.1389*** | -0.0716 | -0.0673*** | **48.48%*** ** |
| | cg03363289 | -0.211* | -0.1501*** | -0.0740 | -0.0761*** | **50.09%*** ** |
| | cg04794268 | 0.294*** | -0.1737*** | -0.0547*** | -0.1190*** | **69.01%*** ** |
| | cg24312537 | 0.298*** | -0.1362*** | -0.0969 | -0.0393* | **27.96%* ** |
| | cg21697381 | 0.293*** | -0.1080*** | -0.0453 | -0.0627* | **59.50%* ** |
| | cg01701207 | 0.229* | -0.1275*** | -0.0921* | -0.0354 | 27.29% |
| | cg09491380 | 0.248** | -0.1321 | -0.0764 | -0.0557*** | 38.72% |
| | cg04657470 | 0.254** | -0.1544*** | -0.1069* | -0.0475*** | **30.10%*** ** |
| P5P | cg15740243 | 0.421*** | -0.1968*** | -0.0846 | -0.1122*** | **57.71%*** ** |

| | | | | | | |
|---|---|---|---|---|---|---|
| | cg21042336 | 0.329*** | -0.2218*** | -0.1773*** | -0.0445* | **19.30%*** |
| | cg04622888 | 0.296*** | -0.2234*** | -0.1668*** | -0.0565* | **24.66%*** |
| | cg00901687 | 0.376*** | -0.2476*** | -0.1968*** | -0.0508*** | **20.34%***** |
| | cg13824270 | -0.367*** | -0.2229*** | -0.1606*** | -0.0623*** | **27.71%***** |
| | cg03363289 | 0.355*** | -0.2131*** | -0.1579* | -0.0552* | **25.54%*** |
| | cg04794268 | 0.434*** | -0.2452*** | -0.0840 | -0.1612*** | **67.69%***** |
| | cg24312537 | 0.405*** | -0.2330*** | -0.1503* | -0.0826*** | **35.35%***** |
| | cg21697381 | 0.351*** | -0.2610*** | -0.1580*** | -0.1040*** | **40.40%***** |
| | cg01701207 | 0.404*** | -0.2239*** | -0.1382* | -0.0857*** | **38.64%***** |
| | cg09491380 | 0.425*** | -0.1971* | -0.0985 | -0.0986*** | **49.40%*** |
| | cg04657470 | 0.333*** | -0.2043*** | -0.1547*** | -0.0496* | **24.59%*** |
| Vitamin B2 | cg15740243 | 0.389*** | -0.1157 | -0.0772 | -0.0385 | 38.39% |
| | cg21042336 | 0.293*** | -0.2546*** | -0.2119*** | -0.0427*** | **16.29%***** |
| | cg04622888 | 0.291*** | -0.2440*** | -0.1754*** | -0.0686*** | **26.06%***** |
| | cg00901687 | 0.268*** | -0.2461*** | -0.2174*** | -0.0287* | **11.41%*** |
| | cg13824270 | -0.333*** | -0.2292*** | -0.1980*** | -0.0311 | 12.16% |
| | cg03363289 | 0.363*** | -0.2291*** | -0.1518*** | -0.0773*** | **32.40%***** |
| | cg04794268 | 0.408*** | -0.1476* | -0.0365* | -0.1111* | **76.79%***** |
| | cg24312537 | 0.317*** | -0.2367* | -0.1967* | -0.0400 | 17.05% |
| | cg21697381 | 0.349*** | -0.0156 | -0.0081 | -0.0074 | 27.99% |
| | cg01701207 | 0.301*** | -0.2276 | -0.1923 | -0.0353* | 13.41% |
| | cg09491380 | 0.361*** | -0.1952*** | -0.1310 | -0.0642* | **31.89%*** |
| | cg04657470 | 0.283*** | -0.2111*** | -0.1617*** | -0.0494* | **23.89%*** |
| | cg25755428 | -0.267* | -0.2469*** | -0.2323*** | -0.0145 | 4.76% |
| | cg06536614 | 0.214* | -0.2476*** | -0.2324*** | -0.0152 | 6.58% |
| Vitamin B12 | cg15740243 | 0.338*** | -0.0784 | -0.0503 | -0.0281 | 11.23% |
| | cg21042336 | 0.308*** | -0.2902*** | -0.2635*** | -0.0266* | **8.47%*** |
| | cg04622888 | 0.304*** | -0.2591*** | -0.2210* | -0.0381*** | **13.08%***** |
| | cg00901687 | 0.284*** | -0.2710*** | -0.2517*** | -0.0192*** | **7.04%***** |
| | cg13824270 | -0.267** | -0.2581* | -0.2346* | -0.0234*** | **8.35%*** |
| | cg03363289 | 0.381*** | -0.2428* | -0.2035* | -0.0393*** | **13.46%*** |
| | cg04794268 | 0.403*** | -0.2850*** | -0.1550*** | -0.1300*** | **46.10%***** |
| | cg24312537 | 0.295*** | -0.2883* | -0.2622* | -0.0261* | 7.93% |
| | cg21697381 | 0.367*** | -0.1839 | -0.1448 | -0.0391*** | 15.61% |
| | cg01701207 | 0.301*** | -0.2783* | -0.2488* | -0.0295* | 9.77% |
| | cg09491380 | 0.350*** | -0.1718 | -0.1306 | -0.0413 | 13.26% |
| | cg04657470 | 0.276** | -0.2470*** | -0.2264*** | -0.0206 | 8.06% |

*$P$<0.05; **$P$<0.01; ***$P$<0.001. Abbreviations: P5P, pyridoxal 5-phosphate; SAM, S-adenosylmethionine.
